# Perspectives of key informants before and after implementing UPSIDES peer support in mental health services: qualitative findings from an international multi-site study

**DOI:** 10.1186/s12913-024-10543-w

**Published:** 2024-02-01

**Authors:** Maria Haun, Inbar Adler Ben-Dor, Cerdic Hall, Jasmine Kalha, Palak Korde, Galia Moran, Annabel S. Müller-Stierlin, Jackline Niwemuhwezi, Rebecca Nixdorf, Bernd Puschner, Mary Ramesh, Ashleigh Charles, Silvia Krumm

**Affiliations:** 1 Department of Psychiatry II, Ulm University, Ulm, Germany; 2 Department of Social Work, Ben Gurion University of the Negev, Be’er Sheva, Israel; 3 East London NHS Foundation Trust, London, UK; 4 Centre for Mental Health Law and Policy, Indian Law Society, Pune, India; 5 Butabika National Referral Hospital, Kampala, Uganda; 6 Department of Psychiatry and Psychotherapy, University Medical Center Hamburg-Eppendorf, Hamburg, Germany; 7 Department of Health Systems, Impact Evaluation and Policy, Ifakara Health Institute, Dar es Salaam, Tanzania; 8 School of Health Sciences, Institute of Mental Health, University of Nottingham, Nottingham, UK

**Keywords:** Peer support, Implementation facilitators and barriers, Consolidated Framework for Implementation Research (CFIR), Low-, middle- and high-income countries, Focus groups

## Abstract

**Background:**

Peer support is an essential part of recovery-oriented care worldwide. Contextual factors have an impact on the implementation of peer support work. However, research has paid little attention to similarities and differences of implementation factors in settings varying by income-level and cultural values. The aim of this study is to assess the factors influencing the implementation of a peer support intervention across study sites in low-, middle- and high-income countries in line with the Consolidation Framework for Implementation Research (CFIR).

**Method:**

6 focus groups with a total of 54 key informants with relevant contextual (organisational) knowledge regarding implementation facilitators and barriers were conducted at six study sites Ulm and Hamburg (Germany), Butabika (Uganda), Dar es Salaam (Tanzania), Be’er Sheva (Israel), and Pune (India) before and 1.5 years after the start of UPSIDES peer support. Transcripts were analysed using qualitative content analysis.

**Results:**

Across study sites key informants reported benefits of peer support for service users and peer support workers as implementation facilitators. At study sites with lower resources, reduced workload for mental health workers and improved access to mental health services through peer support were perceived as implementation facilitators (CFIR Domain 1: Intervention characteristics). The degree of engagement of mental health workers (CFIR Domain 3: Inner Setting/Domain 4: Individuals involved) varied across study sites and was seen either as a barrier (low engagement) or a facilitator (high engagement). Across study sites, adequate training of peer support workers (CFIR Domain 5: Implementation process) was seen as animplementation facilitator, while COVID-19 as well as low resource availability were reported as implementation barriers (CFIR Domain 2: Outer setting).

**Conclusions:**

This study highlights the importance of considering contextual factors when implementing peer support, including previous experience and perceived benefits. Particular attention should be given to organisational benefits such as workload reduction and the allocation of sufficient resources as key drivers in LMICs. In HICs, the potential of organisational benefits for successful implementation should be further investigated and promoted.

**Supplementary Information:**

The online version contains supplementary material available at 10.1186/s12913-024-10543-w.

## Background

Worldwide, peer support is an essential part of recovery-oriented care in mental health services. Peer support workers (PSWs) are people with lived experience who utilise their own experience to help facilitate, guide and mentor another person in their journey of recovery [1]. Previous research showed that peer support has positive effects for service users (SUs) and PSWs, including improved hope, empowerment and social inclusion [1–7]. Numerous peer support programs have been implemented and evaluated in different mental health settings [8–10]. The implementation process of peer support can be facilitated or hindered by several factors. In a recent literature reviews of 53 studies, eight implementation factors were identified, including training for staff and PSWs, role definitions of PSWs, and organisational aspects, which may have a positive or negative impact on the implementation process [11]. Well-known facilitators of the implementation of peer support are education for peer and non-peer staff, a positive attitude of mental health workers (MHWs) towards peer support work, clear role definitions, and recovery orientation of the organisation, while traditional service cultures and inflexible hierarchies were identified as barriers [11–21]. These results are limited for several reasons. Firstly, implementation factors may vary across different settings and countries, and research results that are valid for one setting may not be transferable to another. For instance, a recent qualitative study with 35 MHWs from low-, middle- and high income countries [22] showed that the attitude of MHWs towards peer support also depends on contextual factors, including resource availability and previous peer support experience. Although context seems to play a role for the implementation process, the majority of research on peer support comes from high-income countries (HICs), such as UK [15, 23], Denmark [24], Germany [25, 26] or the US [12, 14]. Only a few studies were conducted in low- and middle-income countries (LMICs) [27–29] or have further compared implementation factors across countries with different income levels [30–32]. Secondly, categories of implementation determinants differed across studies, which makes it difficult to gain systematic knowledge on the implementation process of peer support interventions. In order to address this problem, recent studies and reviews have used implementation frameworks, e.g., the Consolidated Framework for Implementation Research (CFIR) [11, 12, 18, 33]. Further, information about barriers and facilitators of the implementation process should be collected from individuals who occupy influential positions and have a say about implementation, e.g. service managers [34]. Although these experts have relevant contextual (organisational) knowledge, so far research has paid little attention to their perspective.

In order to address this gap, the aim of the current study is to assess the implementation process of the UPSIDES (‘Using Peer Support In Developing Empowering Mental Health Services’) peer support intervention at multiple study sites from the perspectives of key informants with sufficient knowledge of implementation facilitators and barriers at their study sites, guided by the CFIR [35]. The following questions were addressed:

- What are the expectations of key informants towards UPSIDES peer support work in different settings?
- What are the experiences of key informants with UPSIDES peer support work in different settings?

### Implementation framework

The CFIR consists of five major domains, each including various factors with potential impact on the implementation process [35]. Based on pertinent literature [11, 18, 36] and on the best of our knowledge, we selected the following constructs of each CFIR domain for the UPSIDES study context (see Table 1).

**Table 1:** CFIR constructs for the UPSIDES study context

CFIR Domain 1 Intervention characteristics	This domain focuses on key attributes of an intervention which can facilitate or hinder the implementation process. Crucial key attributes for implementation are the benefits of an intervention (Construct: Relative Advantages). In our study, we report key informants’ views on the benefits of UPSIDES.
CFIR Domain 2 Outer setting	This domain focuses on the context surrounding an organisation. Outer Setting includes resource availability or political influences which can hinder or facilitate an implementation process (Constructs: *External Policies and Incentives, Resource Availability)*. In our study, we report external factors influencing the implementation of UPSIDES in different settings.
CFIR Domain 3 Inner setting	This domain focuses on all processes and circumstances which can be found within an organisation. This includes norms, values and basic assumptions of a given organisation (Construct: *Organisational Culture*) or the support of the organisation for the intervention (Construct: *Implementation Climate*). In our study, we report key informants’ views on organisational culture and implementation climate.
CFIR Domain 4 Individuals involved	Different stakeholder groups and their specific characteristics and attitudes towards the intervention can hinder or facilitate an implementation process (*Construct: Knowledge and Beliefs about the Intervention*). For the implementation of UPSIDES SUs, PSWs and MHWs are the most important stakeholders. In our study, we report key informants’ expectations on the attitude of these stakeholders towards UPSIDES as well as experiences with stakeholder groups during the implementation process.
CFIR Domain 5 Implementation process	This domain describes activities which are connected to change processes during an implementation, e.g., engagement of stakeholders (Construct *Engaging*, *Opinion Leaders*). In our study, we report key informants view on training and education for PSWs and MHWs.

## Methods

We conducted focus group discussions (FGDs) (pre- and post-intervention) with key informants across six study sites as part of the UPSIDES study. UPSIDES is a 5 − 1/2-year international multicenter study which aims to scale-up peer support for people with severe mental illness at six study sites in high-, middle-, and low-income countries through mixed-methods implementation research. Key informants were defined as experts who have knowledge of implementing peer support in mental health (MH) services. Potential participants were provided with study information (oral and written forms), informed consent forms (approved by ethics committee) and information sheet about study procedures. All FGD participants were of legal age. Written informed consent was obtained from all FGD participants. The study, including data collection and analysis, was conducted in close collaboration with all six study sites. We followed COREQ guidelines for reporting results of qualitative studies [37] (see additional file 4).

### Setting and intervention

The FGDs took place at six study sites in Ulm and Hamburg (Germany), Butabika (Uganda), Dar es Salaam (Tanzania), Be’er Sheva (Israel), and Pune (India). The study sites differed in types of service provision (inpatient, outpatient, or community services), experiences with PSWs, and organisational readiness before the start of the UPSIDES intervention [18, 38]. A summary of the study context can be found in theadditional file 1.

UPSIDES PSWs are people with lived experience who were trained to utilise their own experiences, along with UPSIDES training and supervision, to help facilitate, guide and mentor another person’s journey of recovery. UPSIDES peer support is delivered for up to 6 months, with a minimum of 3 contacts between SUs and PSWs in a one-to-one or group setting. UPSIDES included service users with severe mental illness. Severe mental illness was defined as illness duration ≥ 2 years and ≥ 5 points on the Threshold Assessment Grid, TAG [38, 39]. PSWs in each site worked with appreciable numbers of individuals with e.g. psychotic features psychotic features, mood problems, and anxiety/trauma related problems. Beyond a common shared core of UPSIDES training and principles the intervention is flexible and thus, can be applied globally in various MH settings (e.g., in- or outpatient) and different countries [10, 38, 40, 41]. In addition to a pragmatic randomized controlled trial assessing the effectiveness of UPSIDES peer support, evaluation of the intervention includes qualitative studies with different stakeholders.

### Recruitment

Study participants were purposefully selected at a local level. Potential participants were contacted in person, by email or by phone. The study aims and FGD procedures were introduced to potential participants during local meetings (e.g., local advisory board meetings). Key informants interested in study participation received an invitation (via a written letter or e-mail) with specific dates and venue. For their participation, participants received a small financial allowance, depending on the study site-based policies.

### Focus group discussions and participants

With regard to data saturation, we follow the concept of code saturation [42]. We assume that sufficient saturation is achieved by conducting 2 FGDs per study site, resulting in a total of 12 FGDs across six sites (six pre-intervention FGDs and six post-intervention FGDs). The pre-intervention FGDs were conducted between December 2019 and January 2020 before the start of the UPSIDES Intervention. The post - intervention FGDs were conducted after UPSIDES PSWs had been working in the organisation for 18 months plus a site-specific intervention pause due to Covid-19 restrictions. The post-intervention FGDs took place between September 2021 and May 2022 while the UPSIDES PSWs were still working at the study sites. In total, 54 participants took part, of whom 36 were female. The average age was 44 years with a range of 22–65 years. Study participants had mixed professional backgrounds including managers, health professionals and policy makers or representatives of service user’s organisations. Characteristics of the focus groups and study participants can be found in Tables 2 and 3.

**Table 2:** Characteristic of focus groups

Study Site	Location		Date		Duration (min)	
	Pre	Post	Pre	Post	Pre	Post
Ulm/Guenzburg, Germany (ULM)	Department of Psychiatry II, Guenzburg		17/01/2020	23/09/2021	60	50
Hamburg, Germany (UKE)	University Medical Center Hamburg-Eppendorf		28/01/2020	09/02/2022	44	104
Butabika, Uganda (BU)	Recovery College, Butabika Hospital		13/12/2019	23/08/2021	105	90
Dar es Salaam, Tanzania (DS)	Muhimbili National Hospital		24/01/2020	16/05/2022	70	60
Be’er Sheva, Israel (BGU)	Community Center, Lod		22/12/2019	19/07/2021	75	60
Pune, India (PU)	Hospital for Mental Health, Pune		30/01/2020	31/03/2022	30	23

**Table 3:** Characteristic of study participants

Study Site	N		Gender		Age range*		Professional Background	
	Pre	Post	Pre	Post	Pre	Post	Pre	Post
Ulm/Guenzburg, Germany (ULM)	2	3	f:1 m:1	f:1 m:2	51–60	51–70	1 Mental health professional in leading position 1 Mental health professional	2 Managers in leading positions 1 Mental health professional in leading position
Hamburg, Germany (UKE)	6	3	f:4 m:2	f:2 m:1	41–70	51–70	3 Service user representatives 2 Managers in leading position 1 Other*	4 Service user representatives
Butabika, Uganda (BU)	6	6	f: 4 m:2	f: 5 m: 1	21–50	31–60	2 Service user representatives 2 Mental health professionals in leading positions 1 Manager 1 Mental health professional	5 Mental health professionals 1 Clinical officer
Dar es Salaam, Tanzania (DS)	7	3	f:4 m:3	f:2 m:1	21–40	41–60	4 Clinical officers 2 Mental health professionals/trainee 1 Other*	3 Mental health professionals
Be’er Sheva, Israel (BGU)	6	4	f:4 m:2	f:3 m:1	31–60	31–60	5 Managers in leading positions 1 Mental health professional in leading position	3 Managers in leading positions 1 Manager
Pune, India (PU)	4	4	f:3 m:1	f:3 m: 1	31–60	41–60	1 Mental health professional in leading position 1 Policy maker 1 Mental health professional 1 Service user representative	1 Mental health professional in leading position 1 Policy maker 1 Service user representative 1 Mental health professional

*Due to data protection reasons no further information can be given here

### Data collection

Focus group data were collected by using two distinct semi-structured topic guides: one for pre-intervention FGDs and another for post-intervention FGDs. The topic guides were developed in cooperation between task leads at the study sites in Germany and Israel (MH, SK, GM, and IAB). The development of both topic guides (pre- and post-intervention) was informed by the CFIR Online Interview Tool as well as by pertinent literature [11, 18]. A preliminary topic guide was presented to the partners at each study site via online meetings and e-mail. The guidelines were then refined (wording, content, comprehensibility). The finalised topic guides (pre-intervention: 4 topics; post-intervention: 7 topics) were translated from English into German (Hamburg/Ulm), Luganda (Butabika), Kiswahili (Dar es Salaam) and Hebrew (Be’er Sheva). Each topic was introduced by a key question followed by sub-questions in case the key questions were not addressed spontaneously. The FGD guidelines can be found in theadditional file 2.

Each FGD was facilitated in the local language by a moderator and an assistant. FGD moderators and assistants had diverse professional backgrounds including psychologists, registered nurses, and social workers. Before the start of the FGDs, the moderators at each site received study-specific online trainings on qualitative research and written instructions on how to conduct the FGDs to enhance comparability. The FGDs took place in line with research hospitality (e.g., drinks/snacks provided) at a place, date, and time convenient for the participants (see Table 2). Due to COVID-19 restrictions, the post-intervention FGD with the key informants in Hamburg (Germany) took place online. A short questionnaire about basic demographic information was given to participants before the start of the FGD. The questionnaire included participants’ gender and professional background. FGDs were conducted in the local language or English (Butabika) and were audio recorded. Shortly after each FGD, field notes were taken on initial thoughts on the main topics or impressions on the interaction dynamics.

### Transcription and translation

Audio files were transcribed verbatim in the language used in the FGD sessions (German, Hebrew, Luganda, Kiswahili, and English). Transcripts were back checked by the moderator against the recording for accuracy. Personal information in the transcripts was deleted or replaced with pseudonyms. To ensure consistent analysis across sites, a bilingual speaker translated the transcripts and field notes from the local language into English before finalisation. Two researchers as part of the UPSIDES translation team checked all translated transcripts to ensure comprehensibility for analysis [43].

### Analysis

We used a deductive-inductive approach to analyse the data following the qualitative content- analysis by Kuckarzt [44]. The deductive approach was guided by the five domains of the CFIR while the inductive approach was used to detect themes emerging from data.

A core group of researchers from study sites in Germany and Israel (MH, SK, IAB, AE) analysed the FGD transcripts. To ensure that the interpretations were informed by a range of perspectives, local research worker teams were involved. The analysis included four steps: (1) Two transcripts including field notes were read and re-read by research workers. To clarify uncertainties, we discussed the transcripts with the local research workers via online site calls (lasting 60 to 180 min). Preliminary codes based on the CFIR constructs were given. Quotes that could not be assigned to the CFIR constructs were discussed considering relevant literature [11, 18] and coded inductively with keywords. A preliminary coding-tree was developed and then applied to the remaining transcripts. Further codes (deductive and inductive) were consecutively added to the coding tree. Memos were used to capture preliminary thoughts and ideas. (3) Themes and sub-themes were reviewed, refined, modified, and structured. (4) To ensure the interpretation is close to the data, the emergent themes were validated with research workers at each site. The pre- and post-intervention transcripts were analysed with slightly different coding schemes. For managing codes, categories, coding trees and memos, we used MAXQDA 2020.

## Results

We report key informants’ expectations and experiences with the UPSIDES peer support intervention corresponding to the five CFIR domains. Results for the pre-intervention FGDs (data collection before the start of the intervention) and post-intervention FGDs (data collection after the start of the intervention) are presented separately for each domain. At the beginning of each results chapter, we provide an exemplary quote that illustrates the findings. A list of quotes exemplifying the five main themes and their subcategories can be found in the additionalfile 3. Quotes were labelled according to the study site Ulm, Germany (ULM), Hamburg, Germany (UKE), Butabika, Uganda (BU), Dar es Salaam, Tanzania (DS), Be’er Sheva, Israel (BGU), Pune, India (PU) and the transcript chapter.

### Benefits of UPSIDES (CFIR Domain 1: intervention characteristics)

*“The hospital managers will benefit, the staff on the ward will benefit, the service users will benefit and the PSWs will benefit. If it’s well managed, it will be a win - win situation.” (BU 45, pre-intervention FGD)*.

#### Pre-intervention FGDs

Key Informants across study sites expected SUs to benefit from peer support. PSWs were seen as role models who strengthen self-confidence and hope of SUs by sharing their lived experiences (BGU 48; DS 35–36; UKE 56; ULM 68). In the FGD in Dar es Salaam, participants anticipated benefits of UPSIDES not only for SUs, but also for their families because of the opportunity to gain knowledge about mental illness and learn about options to support their relatives (BU 44, DS 34). Across all FGDs, participants expected that PSWs themselves would benefit from participating in the UPSIDES training and/or working as PSWs (PU 74; BU 36; ULM 269). For PSWs, providing peer support was discussed as an opportunity to stabilise or improve their mental health (PU 58, UKE 129) and to find work and training opportunities (BU 36; BGU 75). Across FGDs, positive effects on MH services were considered, although less pronounced in comparison to positive expectations regarding SUs and PSWs (PU 74; DS 63; ULM 305). Participants from Butabika and Dar es Salaam expected additional workforce through implementation of peer support and benefits for hospitals (BU 33; DS 41). Additionally, key informants in Butabika expected that MH extend their services, e.g., by PSWs visiting SUs in outer communities (BU 34). Particularly, key informants in the FGDs in Butabika and Pune expected that organisational advantages of peer support for MH services will strongly depend on the successful implementation of the intervention (PU 68, BU 45).

#### Post-intervention FGDs

After the implementation of UPSIDES, participants emphasised that PSWs serve as role models providing hope and support for SUs and their families (BU 28; ULM 55, UKE 73). Especially key informants in the FGDs Dar es Salaam and Butabika reported that PSWs promoted knowledge and awareness for mental illness (BU 30; DS 17), established contact between SUs and hospital staff, and contributed to the prevention of relapses among SUs (DS 11, 120). Furthermore, key informants in Dar es Salaam and Butabika emphasised that PSWs encouraged SUs to adhere to treatment and take medication by sharing their lived experience (BU 28; DS 41, 53). They also described an increase of social support, e.g., improved relationships between SUs and their families and communities (BU 34, 62) and decreased stigmatization (BU 30, 31, DS 46).

After the implementation, key informants across all sites reported various advantages of UPSIDES for the PSWs themselves, including increased knowledge and awareness about mental illness (PU 33, DS 43), and improved self-confidence through the training (UKE 49, 65) or the work itself (PU 23, DS 44). Participants in Butabika and Dar es Salaam reported a higher compliance to treatment and less relapses among PSWs (DS 34–35, 103; BU 27). Key informants assessed UPSIDES as an opportunity for PSWs to find employment or further training opportunities (PU 25–27; UKE 67) which led to increased social acceptance for PSWs (BU 34; DS 64). Participants across study sites expressed mixed views about the benefits of UPSIDES for MH services. A reduction of workload through UPSIDES was reported in the FGDs Dar es Salaam and Butabika, but not in the FGDs Hamburg, Ulm and in Be’er Sheva. In Dar es Salaam, PSWs carried out home visits and provided information to SU and their relatives (DS 49) and, if needed, connected SUs to the hospital, so they could receive treatment (DS 11, 19, 39, 47, 51–53). Therefore, UPSIDES was seen as an additional offer, which brought treatment closer to communities (DS 85). In Pune, PSWs completed recovery plans with SUs, which gave guidance about further treatment (PU 37). Key informants in Dar es Salaam and Butabika reported that through UPSIDES peer support the number of SUs in need of MHWs’ support decreased (BU 28; DS 46–47, 117–119).

### Resource availability, politics and COVID-19 (CFIR Domain 2: outer setting)

*“(…) At the moment, peer support is new to policy makers and nothing much is done to support the intervention. I think it’s the hospital [responsibility] to sensitize and enlighten policy makers about how important peer support is.” (BU 52, pre-intervention FGD)*.

#### Pre-intervention FGDs

Some participants in the FGDs Dar es Salaam and Butabika expressed that mental health education has a low priority in the health system and that peer support is relatively new to policy makers (BU 52; DS 87). Key informants in Pune, Dar es Salaam and Butabika emphasised the urgent need for more resources for the successful implementation of UPSIDES peer support in their organisation. This included the need for space, e.g., to arrange meetings (DS 10–11), working materials, equipment such as identity cards (DS 19, 95), and payment or other incentives for PSWs (PU 143; BU 56–57; DS 17). Key informants in Hamburg were concerned that the implementation of UPSIDES could lead to competition with another longer established peer program in Hamburg. This concern was further emphasised due to resources being insufficient yet for the other peer support program (UKE 129, 133, 143).

#### Post-intervention FGDs

Participants across study sites, except for Ulm, addressed the Covid-19 pandemic as a major unexpected challenge impacting the implementation of UPSIDES. Key informants reported that the intervention plans could not be fully realised due to the pandemic or that SUs could not be reached anymore (PU 51, 108; BU 38, 39). Some participants described that the pandemic had a negative impact on SUs’ and PSWs’ wellbeing, PSWs and SUs could not meet face to face and training courses for PSWs or group sessions with SUs could not be held in person or included only a small number of participants due contact restrictions (BU 20; BGU 29, 48). Offering telephone contact was described as a strategy to provide peer support during the pandemic in Hamburg (UKE 167).

Apart from Covid-19, some key informants reported challenges due to low payment for PSWs or a lack of appropriate places for conducting peer support sessions. According to participants, problems relating to space for peer support sessions could be partly solved during implementation, (BU 32, DS 22, 68). In the FGD Pune participants reported that government policies were supportive for the implementation process. Furthermore, UPSIDES was perceived as an opportunity to implement the national mental health care act to strengthen patient rights and improve their access to social benefits (PU 41, 106, 112). For the sustainability of the intervention, participants in the FGD Butabika expressed a strong need for political support and further funding, especially when MH services more generally are not sufficiently financed by the government (BU 65).

### Organisational culture (CFIR Domain 3: inner setting)

*“Our organisation has 17 years perspective, employing those with knowledge from experience is done without any guidance, it exists in the organisation’s DNA” (BGU 63, post-intervention FGD)*.

#### Pre-intervention FGDs

Key informants described different levels of former organisational experiences with peer support, ranging from no or few former peer support experiences to an established tradition of SUs’ involvement and peer support (BGU 63, ULM 187). Across study sites, organisational support for the UPSIDES intervention was expected to be a key element for the successful implementation of UPSIDES (PU 90–93; BGU 69; BU 12; ULM 166). Key informants in the FGDs Butabika, Hamburg and Ulm expected a recovery-oriented organisation culture as key prerequisite for successful implementation of UPSIDES. For key informants this included an openness of mental health teams towards the perspectives of PSWs as well as SUs’ involvement on the wards or in the organisation (BU 15–16, UKE 26, ULM 14, 26). Additional, some FGD participants discussed the importance of finding PSWs who “fit” well with the organisation and comply with its culture (DS 73; BU 40–42, ULM 162).

#### Post-intervention FGDs

Key informants in Be’er Sheva reported that UPSIDES was implemented without any major problems in regard to organisational settings. According to them, the UPSIDES implementation sites in Israel already valued experience-based knowledge, which facilitated the implementation process (BGU 21, 41, 147). Participants reported that UPSIDES contributed to a further development of the existing concept of peer support work, including the disclosure of lived experience, which was not explicitly expected in mental health rehabilitation organisations in Israel so far (BGU 54–55, 112). Participants in the FGD Ulm and Hamburg (Germany) reported that UPSIDES was implemented largely independent from the existing structures of the MH teams or the inner setting (ULM 27; UKE 103). The structural disconnection between PSWs, MHWs, and a low number of PSWs were introduced as explanations for the small impact of UPSIDES on the organisational culture in Ulm and Hamburg (UKE 115; ULM 65, 78–87). Although UPSIDES was promoted at the study site in Ulm by introducing the intervention in clinic conferences, participants described that UPSIDES was not well-known among MHWs, and as a consequence, hardly promoted by MHWs (ULM 25, 27).

Key informants in the FGD Dar es Salaam did not report any changes regarding the organisational culture, and they referred to the implementation as still being in process (DS 83–84). Participants in the FGD Dar es Salaam and Butabika expressed positive views on the implementation process in general (BU 60; DS 109, 126) and the engagement of MHWs and other stakeholders (BU 19; DS 64).

### Service users, mental health workers and peer support workers (CFIR Domain 4: individuals involved)

*“(…) Some families might not support PSW as they believe mental illness can be treated spiritually.” (DS 84, pre-intervention FGD)*.

#### Pre-intervention FGDs

In Dar es Salaam, participants discussed reservations against PSWs among some SUs and their relatives due to stigma and mistrust. They expected it to be difficult for PSWs to get in contact with SUs and their relatives (DS 84), partly because mental illness was seen as something that is often kept secret by SUs and their families (DS 25). Participants from Butabika expressed uncertainties regarding PSWs, including whether PSWs can uphold professional boundaries between them and SUs (BU 23, 24). Furthermore, key informants in the FGD Butabika expressed worries that PSWs may fail as role models if they relapse (BU 21). Participants in Be’er Sheva considered the risk of PSWs’ relapses as challenging, although not in relation to decreasing PSWs’ credibility. Rather, the discussion was about how to manage relapses adequately in everyday practice (BGU 44).

In some FGDs, participants expected MHWs having reservations against peer support and negative attitudes towards PSWs (DS 69; ULM 10, 18, 86). In their views, PSWs might be seen as a threat to MHWs’ professional status, as they could take over MHWs’ tasks (DS 54, UKE 33). Furthermore, MHWs could perceive PSWs as “additional” SUs than a colleague to work alongside (ULM 55; UKE 39) and in turn increase MHWs workload.

#### Post-intervention FGDs

In general, participants across all sites highlighted that peer support was well accepted among SUs and their relatives (BGU 146; DS 33, 100, 121). However, key informants at the study sites in Butabika and Dar es Salaam reported a few cases in which PSWs received little acceptance from SUs and their relatives (BU 39, DS 102). Key informants in Hamburg and Pune stressed that SUs were not always reliable in attending appointments with PSWs (DS 57; UKE 184–188). Furthermore, participants in Dar es Salaam reported insufficient resources including time or money for transport fees (DS 23). Some participants mentioned that SUs were not sufficiently informed about the UPSIDES intervention (PU 81). Key informants in the FGD Dar es Salaam reported a few cases, where SUs felt insecure during SU’s home visit, e.g., SUs attempts to establish romantic relationships with PSWs (DS 33, 55).

Across study sites, key informants described a high commitment and self-engagement of PSWs working in UPSIDES (PU 27; BU 24, 25; DS 34) mutual support between PSWs (DS 37, ULM 21) and PSWs openness towards MHWs’ advices in case of problems (BU 11). At the same time, participants in the FGD Hamburg highlighted the demands on PSWs (UKE 22, 82), including a pressure to work successfully.

Key informants in Butabika and Dar es Salaam stressed that working together with PSWs and SUs in UPSIDES strengthened MHWs’ positive attitudes towards recovery (BU 32, DS 87–89). In Butabika and Pune, PSWs took part in MH team meetings and provided support to MHWs with daily activities on the wards (PU, BU 45, 50). Participants in Hamburg and Ulm reported fewer contacts and therefore less exchanges between PSWs and MHWs (UKE 103; ULM 21, 39). Key informants in Butabika and Be’er Sheva highlighted that UPSIDES was well received and supported by different stakeholders including managers, hospital administrators, and nurses (BU 19, 41, BGU 41).

### Training (CFIR Domain 5: implementation process)

*“Peer support workers have a change of perspective from client to employee. And that’s a difficult thing. You have to teach it to people.” (ULM 26, pre- intervention FGD)*.

#### Pre-intervention FGDs

Key informants expected a need for training and supervision of PSWs to meet the challenges of providing peer support (PU 122; BGU 32, 36; ULM 26). In addition to the mandatory UPSIDES training and supervision for all PSWs, participants suggested additional training modules or refresher trainings (PU 34–36, 89; UKE 14). FGD participants in Be’er Sheva, Hamburg and Ulm suggested training components focusing on empowering PSWs to use their lived experiences to approach SUs, understand their needs, find their own role as employees in the organisation, and recognise their own stress limits (UKE 51; ULM 299). Participants from Butabika, Dar es Salaam and Pune considered (formal) knowledge on mental illness, organisational values and/or social benefits for SUs as important training contents (PU 127; BU 11, 42; DS 7, 73).

Key informants stressed the importance of sufficient preparation of non-peer staff and mental health teams for the upcoming peer support intervention (PU 84, 86, BGU 30, Ulm 56; UKE 33–34). Some participants emphasised the dissemination of information about peer support as an essential strategy to facilitate acceptance and appreciation of peer support in the organisation (BU 14; ULM 56). Participants in the FGD Be’er Sheva, but also participants in the FGD Dar es Salaam, highlighted leadership engagement as an approach to facilitate acceptance among MHWs in a top-down strategy (BGU 50, 52; DS 68).

#### Post-intervention FGDs

Participants in Butabika and Dar es Salaam emphasised that the UPSIDES training and guidance through supervision was essential in enabling PSWs to provide support (BU 36; DS 11, 12, 16, 62, 64). According to the key informants from the FGD in Hamburg the duration of the training for PSW was too short (UKE 22, 49, 67). Participants in the FGD Pune suggested additional training sessions to empower PSWs, e.g., through psychoeducation on coping mechanisms (PU 61). Key informants reported several activities to disseminate information about UPSIDES and to engage MHWs during the implementation process, including handing out information material and conducting workshops about peer support for MHWs (BU 32, 60; BGU 48; ULM 25).

## Discussion

This study assessed key informants’ experiences with the implementation of UPSIDES peer support in line with the CFIR. To our knowledge, this is the first study comparing the implementation process of a peer support intervention across different MH settings in Europe, Asia, and Africa at two different time points.

### Summary of results

Results will be summarised along the CFIR categories. Regarding **Domain 1** (Intervention characteristics), prior to the start of the intervention, key informants across study sites expected benefits of UPSIDES peer support for SUs’ and PSWs’ well-being. After the implementation, key informants reported benefits for SUs and PSWs as expected. Key informants in settings with low resources availability (Tanzania, Uganda) discussed benefits of UPSIDES more intensively and in more detail, especially regarding the positive impact on MH organisations, such as workload reduction. As to **Domain 2** (Outer setting), prior to the intervention, key informants identified limited resource availability and political support as major barriers for implementation. After the implementation, Covid-19 was viewed as a major barrier across study sites, largely because it hindered or impeded personal contact between SUs and PSWs. In terms of resources for peer support, key informants at study sites in LMICs (Tanzania, Uganda) pointed to more severe challenges compared to study sites in HICs. With regard to **Domain 3** (Inner setting), prior to the start of the intervention, participants across sites emphasised the importance of organisational support. Key informants reported different levels of previous peer support experience and recovery orientation. At some study sites (Hamburg, Ulm), participants reported that UPSIDES was implemented independently of existing structures, while at other sites, UPSIDES was perceived as well-integrated in the organisational setting. Across study sites, participants reported changes through UPSIDES on an individual level, but less on an organisational level. As regards **Domain 4** (Individuals involved), prior to the start of the intervention, participants across all sites expected reservations and negative beliefs about peer support among SUs and MHWs as a challenge for the implementation process. Overall, after the implementation of UPSIDES, participants reported a high acceptance of peer support among SUs, although some doubts regarding SUs’ skills remained. Across study sites, key informants reported PSWs being high motivated to perform their tasks well. Participants at some study sites (Ulm and Hamburg) reported little contacts and therefore less exchange between PSWs and MHWs, whereas key informants at other study sites highlighted a close cooperation between PSWs and non-peer staff. At some study sites, a positive change in MHWs attitudes towards peer support was reported. Finally, regarding **Domain 5** (Implementation process), prior to the start of UPSIDES, key informants assessed training and preparation of PSWs and MHWs as crucial for the implementation process. After the implementation of UPSIDES key informants across sites confirmed the strong need for training and preparation. Particularly, participants in study sites with lower resources (Tanzania and Uganda), reported that the transfer of formal knowledge was an essential training component to enable PSWs to perform their tasks.

### Interpretation of the findings

Across the CFIR domains, barriers and enablers known from previous studies [11, 18] were identified during the implementation process of UPSIDES in diverse MH settings. The reported benefits of UPSIDES peer support on SUs’ and PSWs’ well-being across study sites were consistent with previous research showing that peer support has positive effects for SUs and PSWs including improved hope, empowerment and social inclusion [1–7]. Thus, our study contributes to the existing evidence for the manifold benefits of peer-delivered services for SUs and PSWs not only in HICs, but also in LMICs [27, 30, 31]. In line with previous findings, our study shows some remaining challenges for SUs and PSWs including work-related stress for PSWs [1]. Nevertheless, it appears that these were exceptional cases with only a small impact on the overall assessment of the implementation process. Particular in LMICs it appears that mutual benefits, including reduced workload for MHWs and improved access to MH services for SUs and PSWs, facilitated the implementation of peer support [45]. However, the implementation of peer support as a way to address the care gap in underfunded mental health systems could lead to PSWs adapting their work to fit traditional clinical structures and adopting a medical approach towards mental health [22, 46]. In contrast to participants in LMICs, key informants from HICs (Israel, Germany) reported less potential organisational benefits from peer support. However, a lack of perceived organisational benefits might serve as a barrier for the implementation of recovery oriented services, e.g. by outweighing the costs of the implementation of peer support against the expected benefits [11].

Whether a specific implementation factor was perceived as a barrier or a facilitator, and the importance attributed to it, varied across FGDs and study sites. For instance, previous studies showed that limited or negative experiences with peer support may hinder its implementation [11, 15, 18, 20]. However, in our study limited experiences did not necessarily serve as a barrier to implementation: While at the study site Ulm limited previous experience with peer support had a negative impact on the acceptance of PSWs and thus, the implementation process in general, this was not the case in Dar es Salaam. This might be explained by several reasons: First, in Dar es Salaam, but not in Ulm, MHWs’ have worked closely together with PSWs over a longer period of time, which might have facilitated the acceptance of peer support among MHWs [20]. Second, in Dar es Salaam, PSWs were perceived as valuable supporters by reducing MHWs’ workload. Thus, two facilitating factors (frequent interactions, perceived benefits of peer support work for the organisation) interacted in Dar es Salaam and might have accelerated the implementation of UPSIDES. This suggest that limited previous experiences with peer support work as a potential implementation hindrance could be mitigated in Dar es Salaam, while they remained as barrier in Ulm.

External implementation factors including COVID-19 restrictions and the lack of resources were particularly salient at study sites in LMICs [47]. Key informants assessed resource availability as a key factor for successful UPSIDES implementation throughout all stages [22, 40]. Further external factors included mental health policies within legislative frameworks which were deemed crucial for the implementation process [11, 18, 46]. Across study sites, key informants expressed a strong need for more political support as an important long-term facilitator of peer support.

Our findings on key informants’ suggestions for additional training components are in line with literature reviews on training of PSWs and non-peer staff as a common enabler of the implementation process [11, 18]. However, expectations and suggested training content for PSWs varied to some extent across settings and contexts. Key informants in settings with low resources were more interested in focusing on knowledge transfer in order to address service provision gaps. Thus, consideration of additional context-specific components appears necessary to equip PSWs with specific skills depending on the service context. Nevertheless, emphasising the focus of peer support in sharing lived experiences and highlighting PSWs’ unique skills and knowledge is essential for organisational development towards recovery-orientation [48, 49].

### Strengths and limitations

One strength of the study is the qualitative assessment of the implementation process of UPSIDES peer support across different study sites in Africa, Asia and Europe. We used a widely accepted implementation framework to capture information about facilitators and barriers in the implementation process on two measurement points. However, there are some limitations. First, we used a purposive sampling strategy, and our findings are based on a small sample of key informants at six study sites. Thus, our findings cannot be generalized to key informants’ experiences with peer support interventions at the study site. Social desirability might have also influenced key informants’ statements. In addition, the perceived importance of different implementation factors might not necessarily correspond with the “real” situation at the study site [50]. Secondly, most FGDs were translated from the local language into English without backwards translation and the transcripts were analysed mainly by researchers in Germany. Thus, it is possible that certain cultural aspects affecting key informants’ views on the implementation process were overlooked. Third, in some cases quotes could not be clearly allocated to the CFIR domains because they referred to more than one CFIR or did not fit in any domain. Further, CFIR domains may not reflect differences between countries and cultures appropriately, e.g. not accounting for the specific administration of health systems or community characteristics influencing the implementation process.

## Conclusion

Beyond the broad consensus on the range of benefits of peer support on the individual level, this study confirmed the importance of considering contextual factors when implementing peer support. Illuminating the complex interplay between single facilitators leading to different outcomes at the local level of MH Services is important. Particular attention should be paid to organisational benefits such as workload reduction and the allocation of sufficient resources as key drivers in LMICs. In HICs, the potential of organisational benefits for successful implementation should be further identified and promoted. In study sites with less peer support experiences, establishing organisational structures enabling frequent contacts between PSW and MHWs is crucial to increase cooperation and decrease reservations. Further recommendations for successful implementation of peer support in MH settings include context-adapted training modules addressing specific needs of local MH service and the establishment of supportive legal frameworks.

### Electronic supplementary material

Below is the link to the electronic supplementary material.

Additional file 1: The context of the UPSIDES study sites

Additional file 2: Topic guides

Additional file 3: Participants’ quotes

Additional file 4: Consolidated criteria for reporting qualitative studies (COREQ) 32-item checklist

## Data Availability

All transcript fragments which informed the analysis presented in this publication are included within the paper and its online supplement files. Full transcripts are not publicly available due to their containing information that could compromise the privacy of research participants.

## Abbreviations

HICs: High-income countries
LMICs: Low- and middle-income countries
FGD: Focus group discussions
SUs: Service users
MHWs: Mental health workers
PSWs: Peer support workers
MH service: Mental health service
UPSIDES: ‘Using Peer Support In Developing Empowering Mental Health Services’
CFIR: Consolidated Framework for Implementation Research. ULM:Ulm (Germany)
UKE: Hamburg (Germany)
BU: Butabika (Uganda)
DS: Dar es Salaam (Tanzania)
BGU: Be’er Sheva (Israel)
PU: Pune (India)
