# Supplementary material for: Perspectives of key informants before and after implementing UPSIDES peer support in mental health services: qualitative findings from an international multi-site study
Source: BMC Health Serv Res. 2024 Feb 1;24:159. doi: 10.1186/s12913-024-10543-w (PMC10835950; doi:10.1186/s12913-024-10543-w)
Supplement: Supplementary file 2 — Additional file 2: Topic guides [file 12913_2024_10543_MOESM2_ESM.docx]

**Additional file 2: Topic guides**

**Topic guide pre-intervention**

| Topic | Question |
| --- | --- |
| Implementation of Peer Support | From your point of view what is the most important ingredient to implementing  UPSIDES Peer Support at [name of organization] successfully?  Additional question:   - *What has been the biggest challenge during the implementation of UPSIDES so far in [name of organization]?* - *What kind of challenges are you expecting?* - *In your opinion what´s the biggest benefit of UPSIDES Peer support for [name*   *of organization]?*   - *Was it necessary to adapt UPSIDES Peer Support to local conditions? If yes, what were the most important adaptations?* - *What are the most important aspects for long-term implementation of UPSIDES Peer Support at [name of organization]?* |
| Individuals involved | How do you think UPSIDES Peer Support will be accommodated/accepted by  -service users  -mental health workers  -managers?  Additional question:   - *Which group of people will benefit through UPSIDES Peer Support?* - *Which group of people will have difficulties with UPSIDES Peer Support?* |
| Inner Setting | You are familiar with the conditions/situation at [name of organization].  How well does the UPSIDES intervention fit with the values and norms within the organization?  Additional question:   - *How do you think your organizations’ culture (general beliefs, values, assumptions that people embrace) will affect the work of the PSWs?* - *Do you expect changes due to UPSIDES in [name of organization]? If so, what Intervention changes do you expect?* |
| Outer Setting | What role do factors outside [name of organization], such as laws and regulations, play in implementing UPSIDES peer Support locally?  Additional questions:   - *What kind of local, state, or national performance measures, policies, regulations, or guidelines influenced the decision to implement UPSIDES peer support?* - *What kind of financial or other incentives influenced the decision to implement UPSIDES Peer Support?* |

**Additional file 2: Topic guides**

**Topic guide post-intervention**

| Topic | Question |
| --- | --- |
| Implementation Process | When you look back to the implementation processes of the UPSIDES intervention today – what is your overall opinion on the implementation processes?  Additional questions:   - *What went well in the UPSIDES implementation process?* - *What went not so well in the UPSIDES implementation process?* - *Was there anything you have been surprised about regarding the UPSIDES developments implementation process?* |
| Impact of UPSIDES peer support | In your opinion, what is the overall impact of UPSIDES?  Additional questions:   - *In your opinion, how did UPSIDES impact on SUs/on mental health teams?* - *If you look back: What is the biggest benefit?* - *If you look back: What is the biggest challenge?* |
| Facilitators and Barriers | Which factors do you assess as most important for a successful implementation of UPSIDES so far in [name of organization]?  Additional questions:   - *Which factors/resources in the institution helped most to implement UPSIDES peer support?* - *What made it difficult to implement UPSIDES peer support in your institution?* |
| Characteristics of Intervention (Adaptation) | Sometimes it is necessary to adapt a program to the local context. Are you aware of adaptations of the UPSIDES Peer Support since the start of the intervention to local conditions? If yes, what were the most important adaptations? |
| Inner Setting | Has UPSIDES contributed to changes in [name of the organization]? If yes, to which ones?  Additional question:   - *How do you think was your organizations’ culture (general beliefs, values, assumptions that people embrace) affected by the work of the PSW?* |
| Outer Setting | Which external factors [name of organization] influenced the UPSIDES implementation process most?  Additional questions:   - *In your opinion, what role do external activist movements play for the successful implementation of UPSIDES peer support work [in your organization]?* - *Were there other peer programs in [name of the organization] at the time of the peer support? If yes, what influence did this have?* |
| Implementation Process | In summary, do you think the implementation of UPSIDES was successful in [name of organization]?  Additional questions:   - *What is the most significant evidence for the successful implementation of UPSIDES in your organization? Can you give an example?* - *What is the most significant evidence for not being fully successful with the implementation of UPSIDES in your organization? Can you give an example?* - *What do you think about the further development of UPSIDES in your organization? What do you wish for?* - *How do you see the chances that UPSIDES Peer Support will still be in [name of the organization] e.g. in 2 years? To realize this - what has happened so far/what would need to happen?* |
