# Supplementary material for: Perspectives of key informants before and after implementing UPSIDES peer support in mental health services: qualitative findings from an international multi-site study
Source: BMC Health Serv Res. 2024 Feb 1;24:159. doi: 10.1186/s12913-024-10543-w (PMC10835950; doi:10.1186/s12913-024-10543-w)
Supplement: Supplementary file 3 — Additional file 3: Participants’ quotes [file 12913_2024_10543_MOESM3_ESM.docx]

**Additional file 3: Participants’ quotes**

Quotes are listed according to the focus groups pre- or post-intervention) and the five CFIR domains. Additionally, quotes are labelled in regard to the study site Ulm (Ulm) and Hamburg (UKE), Germany; Butabika, Uganda (BU); Dar es Salaam, Tanzania (DS); Be’er Sheva, Israel (BGU); and Pune, India (PU)

Benefits of UPSIDES (CFIR Domain 1: Intervention characteristics)

## ***Pre-intervention FGs (CFIR Domain 1: Intervention characteristics)***

“We already know that those coping with mental illness are lonely and withdrawn, not all.. Once the program starts it can unify and give.” (BGU 48)

“P4: I was thinking the benefit which the service users will receive is that first it will help to reduce stigma especially patients’ self-stigma some patients may be saying that they are not capable of doing something (…) so we are going to help service users accept themselves and will become testimonials to other patients and provide more knowledge on mental illness and help others. So if we help them they will be free, confident and will be able to assist other people in their recovery journey.

P6: The benefit that I see is that it will help build self confidence among patients and improve self-care by knowing that there are other people who went through this and they have been able to overcome.” (DS 35-36)

“(…) the client, in that moment [sees]: ‘He or she is not so different from me and does this anyway and (…) I can do that too, I don't have to do exactly the same thing, but according to the motto: That doesn't mean that you have to be perfect to be able to do anything at all.’ So it can sometimes be empowering for yourself to see where others have difficulties. Without that now means looking down on them or anything.” (UKE 56)

“They [PSWs] are not therapists. They're here to say [to the SUs], ‘hey, look at me, let’s have a coffee, I could leave the hospital context. I'm the example that it works.’ (…) I don't have the golden way, but (…) I'm the proof that it [recovery] works’ (…).” (ULM 68)

“I think the service users will benefit more because they will be empowered to keep their wellness by reducing relapses. The family will benefit and the whole nation at large.” (BU 44)

“Another thing is that the service user’s relatives will also learn more about mental illness when they take part in some of the PSW sessions and how they can support service users. Also it will help them with the feeling that they are burdened by taking care of their relatives alone they will know that they assisted by health care providers and peer support workers that will help the patient recover and they would gain more knowledge. For example during the pilot the service user would come with her daughter during the peer support sessions and we became connected and we are still communicating until today although the pilot phase has ended.” (DS 34)

“PSVs [peer support volunteers] themselves are getting benefited, few of the patients are getting benefited because of the [name of the group] concept which is really working well as. I totally agree with that.” (PU 74)

“More so, it can be you are providing employment opportunities to PSWs because through empowering others, they get occupied instead of being redundant at home. They will be having a job, can go and work, can buy medicine in case it’s not available in the hospital.” (BU 36)

“(…) you said before that this experience of illness is a treasure, right? I think it's a very important story in peer support work. To say that I have an experience that only I can offer. (…) I would like to see people with psychiatric experience who have learned to live with it, who perhaps learn to live with it themselves through a peer support story like this, whoa, that's something I can show here. This is not just crap. I used to be a stigma, I used to be in the district hospital 1, but, no, that's a treasure that I learned to deal with this experience well. And I can take some of that treasure and give it to others.” (ULM 269)

“Benefits if we see at the level of PSVs [peer support volunteers] then peer support volunteers stay stable with medicines and come with the symptoms. So they, with their interest, stay engaged with other patients. So their thoughts are going in a more productive way (direction). And simultaneously they also receive motivation support and motivation counselling from here. So the self-esteem and self confidence level that is needed gets better, that’s one benefit. The second thing is that, the ones who sustain, their social and community integration becomes easy for them as well as for us.” (PU 58)

“Many people do not want to work with it [as PSW] at all but use it [the training] for their own benefit. I think it's very, very justified.” (UKE 129)

“More so, it can be you are providing employment opportunities to PSWs because through empowering others, they get occupied instead of being redundant at home. They will be having a job, can go and work, can buy medicine in case it’s not available in the hospital. (BU 36)

“My peer taking part wants to study social work. It is empowering her that she can. A bridge to being more of a professional in this field. A belief in what the individual can achieve a platform that encourages.” (BGU 75)

“So benefit for the hospital is yet to be achieved. Right now, no benefit for the treatment part for the doctors.” (PU 74)

“I think its service users who will benefit more because the management is there to help service users, mental health workers and now peer support workers are also there to help service users so their healing process will be fast compared to the past (…) The department will also benefit from providing good services but the service users will be benefiting more.” (DS 63)

“I think it [peer support] is very helpful overall. I can't say whether the individual institutions will make so much profit from it that it will pay off. But at the end of the day, if you stick to it, it will help. Because it already does. The peer support that takes place here is an important factor. It's just not being understood yet.” (ULM 305)

“Peer support will help in bridging the human resource gap since they have their special roles in areas of psycho social support.” (BU 33)

“I think that the hospital and patients will experience recovery within a shorter period of time. I think the peer support program will help to someone to be in recovery within a short period, apart from receiving support from the psychologist and occupational therapist he/she will also receive support from us so it will reduce the time in which the patient is in acute condition” (DS 41)

“Services are brought nearer to the service users especially into the community whereby PSWs in a given community can easily identify and refer for further management” (BU 34)

“Per se, the hospital is not getting any benefit. I will say that hospital will get benefited if we really make our purpose that PSVs [peer support volunteers] can become instrumental for psychoeducation or for community awareness or for helping patients in getting social benefits, which we had planned but has not been done till date.”(PU 68)

“This is an intervention where there will be a win - win outcome. The hospital managers will benefit, the staff on the ward will benefit, the service users will benefit and the PSWs will benefit. If it’s well managed, it will be a win - win situation. The hospital managers will get good publicity because of using their own people; the staff workload will be reduced, PSWs will get routine in their life, empowered and feel recognized. The community will benefit because PSWs will be knowing a lot and will create awareness, since they will be well conversant with mental illness. “(BU 45)

## ***Post-intervention FGs (CFIR Domain 1: Intervention characteristics)***

“My opinion on how UPSIDES has actually impacted on the service users has been very evident that the participants that have been followed…, we’ve had preliminary report from the families that these clients are doing much better than before… (…) they have been given hope…, even the families.” (BU 28)

“So it is safe for the clients to see that this is someone who also had a difficult time, who was also ill, who has managed it, who understands me, who knows exactly what I am talking about and knows the feelings because he has gone through it himself. I think that on the one hand they can identify with that and for the professionals it is also like that, that you have this feeling that is a different access that the peers have to the patients. That's a different approach and that's also quite interesting when you talk about a patient, what hypotheses the peer support workers have, why is it like this and like that? And simply another input.” (ULM 55)

“(…) I think - no matter if UPSIDES or something else - that is the, yes that is what can be good for people who are accompanied. That they have the feeling, I also have a chance.” (UKE 73)

“To me, I view UPSIDES project to have increased awareness to service users and to the family as well. Their families are directly involved in their management, they support them [SUs] more than it has been before; and this has also reduced on the relapses (…)” (BU 30)

“What I see has gone well is getting the peer support workers so as to provide education to others who also have mental illnesses. It has an impact to the peer support worker who received the training and the service user who has received the intervention, the service user is educated about mental health challenges.” (DS 17)

“And the evidence which shows that things were going on well is that some of our patients who were hospitalized regularly the number of hospitalizations had decreased. When the peer support workers saw some signs that the service users were showing they used to consult us, we communicate with the relatives so that they can bring the service user to the hospital, we request for an appointment from his doctor, so some of the service users received treatment early before they relapsed. So, I think that the implementation process was done well.” (DS 11)

“I don’t have the evidence in figures but I have examples. There are some of our patients who were chronic or were relapsing frequently we don’t see them anymore, those who have been enrolled in the project we don’t see that is why we are saying that it is something which has helped them. And sometimes when you meet the service users, they tell you that UPSIDES peer support has helped them. There are some of our patients who are close to the peer support workers, so communication helps.” (DS 120)

“(..) they [SUs] are more compliant (…) the biggest benefit, it’s the compliance from the participants …, because their level of compliance as opposed to what they have been going through …, they are better supported to gain insight.” (BU 28)

“The overall impact is that stigma is reduced because the service users receive the services from PSW who have lived experience of mental illness, they understand the challenges on the use of medicine more than a professional, because a professional has just studied but has never taken the medication, but if this service user is told by a person who has lived experience, who is using the medicine so even if he is told that those challenges will come to an end he will understand well compared to when he is told by another person. So, the major impact which I have seen from the service users is the possibility of taking medication, attending clinic, because other have lost hope. So, reduction in stigma can be a big impact on the service users.” (DS 41)

“The biggest benefit is to reduce relapses and stigma and treatment adherence; those are the three main benefits. To help with treatment adherence, to reduce stigma, to reduce relapses and to increase knowledge on mental health conditions to the PSW. It has boosted self-esteem and confidence of the peer support workers, it has boosted their dignity they see that they have value and a person who has function in the community. It boosts all those things which are important on mental health state, being functional or beneficial in the community.” (DS 53)

“UPSIDES has empowered some of the PSWs in the community and some of them are called doctors…, so they have created a relationship with the families.” (BU 34)

“Some of the evidences that the project has been a success…, to me the most vivid one has been the controlled relapses and the increase in family and community involvement. This project has really empowered families.” (BU 62)

“(..) they [SUs] can easily identify the [psychological] triggers and know how to avoid them and this has improved on their productivity because once the relapses are controlled and someone is accepted in the family and the community, then they can be productive ..., because their level of functionality increases and their contribution is appreciated.”(BU 30)

“And also the other issue is controlled stigma …, most of our participants in UPSIDES could complain of the stigma…, which would originate from their own homes..., with the neighbours and the community at large. But because PSWs could move up to their families …, and they happen to witness that one on treatment can be productive…, so stigma has also been somehow reduced or controlled.”(BU 31)

“I think that when you see your patient is self-aware, she cares for her health then it is positive to us that is the main thing. But also if you see that they are not dependent and it was my wishes that my patient should reach a level where she is independent we are happy. So I think that we have a big impact on the community to remove dependency.” (DS 46)

“And also the PSVs [peer support volunteers] themselves because it helps them understand their illness better because they have been exposed to number of other patients who have diverse symptoms.” (PU 33)

“Also education on mental health conditions, they [PSWs] have been aware.” (DS 43)

“I think the [UPSIDES] program is actually good. So I think the [UPSIDES] training program is actually good too. In fact, I have the feeling - and I know this from people I have accompanied - that they dared to do an X program [another peer training program] afterwards.” (UKE 49)

“Yes, well, I think that it is also that you get an upgrading, that is, through the experience that you have as someone who experienced psychiatry. That you can apply it in a useful way that I can apply it in a useful way. And then a few good tips on how to do it. So just this, how important this expert experience actually is, you learn that again.” (UKE 65)

“So, overall experience is that it [UPSIDES] helped the PSVs [peer support volunteers] in gaining their self-confidence back.” (PU 23)

“To a large extent it has boosted their confidence and their stigma has greatly diminished, it has also increased their awareness on the disease because when you become a peer support worker it means that you are learning, you are looking for the right information so it has increased their knowledge on mental illnesses. When you become a peer support worker automatically you learn so that you can deliver the education to others, it increases knowledge on mental illnesses.” (DS 44)

“P1: Most of them are our patients whom I know and who were relapsing from time to time, what surprised me is that during all that time when thy were performing their roles as PSWs they had not relapsed. I am surprised why they haven’t relapsed, is it the work that has motivated them. There are people who I had expected to see them being hospitalized but we haven’t seen them here. So the thing which surprised me is that there were no relapses for the PSWs.

P2: They had consciousness, they did something which was unexpected they have done wonders. You find that when she talks to a patient or service user you wonder that this is a patient but talks as a mental health professional and as my colleague said that when they were confident even their behaviour changed. In the past they were people who weren’t adhering to medication but they started to adhere to medication because they thought that if they don’t take medication they will relapse and won’t be able to conduct intervention sessions. So, they adhered to their treatment, they were smart so that they can be presentable to their clients and even the community so that they can see that this person is really a health worker, it changed them very well.” (DS 34-35)

“The peer support workers accepted themselves, even when they are with their clients they did not deny that they are also clients, they were very motivated to the extent that they didn’t care if the service users knew that they are also patients. And when they went to the community they said that they are also patients, it is something which built them.” (DS 103)

“UPSIDES has benefitted participants and PSWs…, it has empowered them to cope up with life on an individual basis. It has given them a shoulder to stand firm on what they need, what they believe in and to also act as any other person in life. This was proven by the pandemic that we have gone through…, most of them especially the PSWs would have relapsed…, because this was a situation that did not segregate who is mentally stable and who is mentally sick…, but if I look through the reviews, they have proved to be stable. So I give a credit to UPSIDES…. that it has empowered these peoples to cope up with life challenges.” (BU 27)

“So one thing was that the PSVs [peer support volunteers] themselves had good confidence after they got enrolled in the program. Second thing is like, it was road to rehabilitation to them (...) So maybe after this, you know, they can have a vocational rehabilitation because you know, they have already started working here as part of the staff although they are volunteers and we are not giving them huge incentives. (PU 25-27)

“(…) whoever has developed such self-confidence from this further training or from doing that, that they might then go one step further into the qualitatively - I say it deliberately in quotes – qualitatively higher education or further training (…)” (UKE 67)

“UPSIDES has empowered some of the PSWs in the community and some of them are called doctors…, so they have created a relationship with the families.” (BU 34)

“I think the workshop and training to the peer support workers enabled to strengthen them. I think the training helps because this person was facing stigma of his illness, maybe the community rejected him before so if he doesn’t [didn’t] receive training it might be a challenge.”(DS 64)

“As health care providers it has reduced the burden of providing education, since the number of relapses drops then there are few caseloads. It has helped in providing education, instead of us doing it they are the ones who deliver the education so it is support to us. Also, when the number of relapses decrease we know as an institution there are issues related to medication, if there are few relapses the dosage decreases so it is a benefit to the hospital in enabling patients to receive medicine or treatment. If there are many relapses it means that the dosage which the patients receive increases which will be costly for the hospital or institution or the donors. Of course, the government is the one who provides medicine, so it is a benefit to health care providers.” (DS 49)

“And the evidence which shows that things were going on well is that some of our patients who were hospitalized regularly the number of hospitalizations had decreased. (DS 11)

“One is that the number of relapses has decreased compared to the past before the project started because before this project started, I used to see many of our patients have been hospitalized, there were regular hospitalizations among our patients, after this project started I have seen that the number of hospitalizations has decreased, even the number of appointments to me had decreased.” (DS 19)

“As I mentioned earlier that there are some patients who are in this program whom I used to see frequently before the program started but now I haven’t see them for a while to the extent that some of the nurses I am working with when they saw some patients they were inquiring if the patients can’t be enrolled in the program because they have seen that for those patients who have been enrolled in the study their number of hospitalizations has decreased, so they are saying that the project is good and has benefited many. So I have seen that UPSIDES project has a positive impact.” (DS 39)

“What I see is that it reduces my workload especially for the patients who were coming regularly, it has reached a point where we are saying that there are few cases of hospitalizations and maybe because it has been contributed by UPSIDES project. So, I see that if there are such programs it will make people to be self-aware, people to know the importance of attending clinic and taking their medication and being productive. Our workload has also decreased.” (DS 47)

“P1: The workload is reduced, they help to provide education and they reach further than us, for us a person has to come to us but for the peer support worker he goes to visit the person wherever he is. So, for us it helps in identifying someone who is at risk of relapse because many of the PSW when they saw clients who had warning signs they used to refer them to the hospital early but in the past before the intervention many of the service users weren’t coming on time and they were relapsing regularly. By being close to us the peer support workers help in identifying my client who is not well and recommend her to see a psychiatrist or psychologist so that they can discuss about her challenges and solve them early before the big challenge occurs.

I: Okay. If you look back, what is the biggest benefit of UPSIDES intervention?

P1: The biggest benefit is to reduce relapses and stigma and treatment adherence; those are the three main benefits. To help with treatment adherence, to reduce stigma, to reduce relapses and to increase knowledge on mental health conditions to the PSW. It has boosted self-esteem and confidence of the peer support workers, it has boosted their dignity they see that they have value and a person who has function in the community. It boosts all those things which are important on mental health state, being functional or beneficial in the community.” (DS 51-53)

“What I see is that there are community services which the department is supposed to provide, it seems that these services were not so active but UPSIDES intervention was like a community-based intervention, they used to visit the service users and deliver the intervention in the community. It has helped because the community sees that they have been visited, although those who were providing the service are also clients but the education they were giving was the one which I could also go and give. So, the community sees that they have started to be visited at their homes, it has helped a lot.” (DS 85)

“(…) Third thing, if we see how it benefits the hospital. So benefits to the hospital include that now we have a recovery plan for almost all patients. We can get guidance from this that what should be done for an individual patient.” (PU 37)

“And on mental health team I would generally say that the more the patients are compliant and take a lead role in managing their mental health as a result of the impact of UPSIDES and peer support, the team gets a relief on such many cases of relapses (…)” (BU 28)

“P3: I think that when you see your patient is self-aware, she cares for her health then it is positive to us that is the main thing. But also if you see that they are not dependent and it was my wishes that my patient should reach a level where she is independent we are happy. So I think that we have a big impact on the community to remove dependency.

P2: What I see is that it reduces my workload especially for the patients who were coming regularly, it has reached a point where we are saying that there are few cases of hospitalizations and maybe because it has been contributed by UPSIDES project. So, I see that if there are such programs it will make people to be self-aware, people to know the importance of attending clinic and taking their medication and being productive. Our workload has also decreased.” (DS 46-47)

“P1: The evidence is that my main interaction was with peer support workers rather than service users I don’t meet with them often. So my evidence is that my client whom I linked to this project I think that from when the project started she has just made an appointment with me one or two times compared to the past she used to come from time to time to ask for appointments, there were many crises. So my evidence is that I have a client who is a peer support worker, I was seeing her but since the project started it is only once where she had signs of relapse. When did the intervention start?

M: 2020.

P1: 2020 until now 2022 is two years, I noticed that crisis just once but before the UPSIDES peer support intervention started she used to come every month with different challenges.” (DS 117-119)

# **Resource availability, politics and Covid-19 (CFIR Domain 2: Outer setting)**

***Pre-intervention FGs (CFIR Domain 2: Outer setting)***

“(…) at the moment, peer support is new to policy makers and nothing much is done to support the intervention. I think it’s the hospital to sensitize and enlighten policy makers about how important peer support is. Otherwise at the moment, nothing much is done, it’s something our directors have to fight for.” (BU 52)

“I think the education policy, to educate the society about mental illness because here mental illness is not prioritized like other illness such as cancer, HIV.” (DS, 87)

“P1: I think when we start peer supporters should have their own place where they can meet on their own and with service users without interruptions because sometimes the place that we usually meet has other people working there at the same time that we want to have our sessions.

P2: I would like to stress more about that PSW having their own place of meeting were they can do their personal activities and meet with their service users because sometimes it could be raining or too sunny and sitting outside will not be the good.” (DS 10-11)

“Another is working equipment’s such as IDs should be given to PSW so that they can be identified in different places that they will visit during the time of the study and another thing is that PSW should be given certificates after their training so as to help them in the future.” (DS 19)

“I think working materials/equipment’s things that we will need during the work.” (DS 95)

“(…) at the level or PSVs [peer support volunteers]...it is difficult to get people at such a low pay.” (PU 143)

“P6: If PSWs are paid something, they get empowered, if they could get a salary it would motivate them.

P2: Another way if incentivizing PSW is by making their life easy. Could get an in-kind contribution for example private services can be given at a lower rate, lining up at Out Patient Department for medication could be lessened so that they get services faster.” (BU 56-57)

“I think peer supporters should be assured that they will be offered financial support to enable them in their work. It could be a contract or any other way but they should have support system they should know for sure that on this day they will be given the money or not because if they do not have money for transportation it means that they will not work or they will work in difficult situations and a PSW cannot conduct a session with the service user.” (DS 17)

„I see the competition's problem, since it [the other peer provider organisation] is in the same city too. That doesn't mean I like it and it doesn't mean I'm in it. I think the more peers the better (…)” (UKE 129)

“But first we have to stop thinking: some [peer providers] are better than others here and they get paid less. We all get paid very badly (…)” (UKE 133)

“(…) I don't think it has to be competition [with other peer support providers]. I think it is more like what you said to really open that up and say: You network.” (UKE 143)

***Post-intervention FGs (CFIR Domain 2: Outer setting)***

“So that [financial remuneration for PSWs] is the biggest challenge from what I see...Second was the Covid. Covid had a lot of, you know, setbacks. Because of Covid many plans were not fulfilled.” (PU 51)

“I – Which external factors (…) influence the implementation of UPSIDES project the most?

(…)

R4 (…) The biggest external factor was the COVID.” (PU 102-108)

“(…) what made it difficult is the relocation of participants due to the pandemic. Most people were destabilized financially and a lot of adjustments had to be made…, which involved moving from urban to rural areas. So this relocation made it very difficult for the participants to be reached and for UPSIDES program.” (BU 38)

“And then of course I cannot leave out covid19 still…, the Covid restrictions have made things really hard; but having said that…, we tried to maneuver around and continued with our work.” (BU 39)

“(…) the PSWs were affected as well in a way that as a result of Covid-19 ; their welfare was affected and of course also affected their engagement with the participants [SUs]. Many of them had issues to cope with the financial constraints, the restrictions involved…, and many other concerns that came up.” (BU 20)

“I can say that like with any guidance of any group the moment you limit them to eight plus two because of COVID regulation, we have a problem of dropouts in every [recovery] group. We always prefer there will be at least ten participants in groups because there is the option of dropouts. Gladly this group did hold on, it held enough participants to maintain the group, but there is no doubt it’s a challenge that is built-in the COVID limitations. It is still a million times better than groups via Zoom and it is better ending up with three or four people than not having it at all.” (BGU 29)

“(…) looking back when you go into COVID again and another quarantine, so you don’t meet and then come with masks, don’t come with masks, and some are isolated, some are sick, so it’s hard (…)” (BGU 48)

“And you asked for Corona, right? But that has not only been a stumbling block for UPSIDES... How shall I say it, it has been a stumbling block for all people. Corona is simply a crazy challenge, and it has led to the fact that the people who have offered help have mainly made contact by telephone. Or at least it was completely unclear whether it was possible to meet or not. Then you put on this mask and don't really see yourself. These are all things that we have become accustomed to in a certain way, but they are horrible. And as far as fellow human beings are concerned, something has been lost that we don't really know what it is yet. But that doesn't only affect UPSIDES. It affects all people and... But that has certainly made it more difficult.” (UKE 167)

“Then also, to me the biggest challenge in UPSIDES has been about remuneration of participants [PSWs and SUs] (…) the project did not have enough funds.” (BU 32)

“There was a challenge on the issue of the space/area to conduct the intervention sessions because you find that this area which they used to conduct the sessions there are times when other activities are going on so we tried so much to squeeze ourselves but it was not that calm. So the environment was not that much good to them.” (DS 22)

“The use of our offices, if it wasn’t available then it would have been a challenge. So they might need a place to meet and get access to it, although the space was limited but it was available. In the future we need to establish an office or special place within our institution for the project. For the space which we have now, the peer support workers might come and find that there is another activity which is going on so you have to wait for it to end.” (DS 68)

“So that will help the patient, the hospital and the PSV [peer support volunteer] themselves for increasing their knowledge and getting the benefits. And you know, implementing the MHCA [Mental Health Care Act]. So, these are the benefits as I see.” (PU 41)

“So what external factors can affect is the policy of the government. So, the policy of the government is right now supportive for this project.” (PU 106)

“So if it is supportive or non-supportive, if it is conducive or non-conducive all these [implementation] factors are also affecting. But right now, if you look at the biggest parts, one is COVID which took it towards to negative part and the second is the government policy which is positive.” (PU 112)

“(…) when you come to sustainability, to tell you the truth, I don’t see any future for UPSIDES project…, there are no chances…, because for me I think once the study is done and the funding is done, and then it will end there. But if hospital maybe can take over to continue supporting PSWs do their activities, it would work which I know…, because I am part of the hospital team …, that it’s very difficult …, funds from the government are not there…, because even hospital departments are not fully funded, then what do you think about peer support work which has just come in…, it might not work. So for I think Butabika and Uganda at large being a low developed country, we need continuous funding if this is to work…, otherwise we will acquire knowledge and skills, we will know what is needed to be done…, and it will remain on paper…and that will be the end.” (BU 65)

# **Organisational culture (CFIR Domain 3: Inner setting)**

## ***Pre-intervention FGs (CFIR Domain 3: Inner setting)***

“Our organisation has 17 years perspective- employing those with knowledge from experience is done without any guidance- it exists in the organisation's DNA and we want to continuously give opportunity. It's informal and about seeing eye to eye and allows a good feeling. The organisation as an organisation is very compassionate and allows it, even if its not centralised even though its important.” (BGU 63)

“X [Name of a former peer Intervention] was implemented here. It just didn’t work well. The experience is of course still in the background. (ULM 187).

“I: (…) what according to you are the factors due to which (…) peer support has sustained here?

P1 : Because of the commitment of the staff

I: Commitment of the staff...right

P1: Okay, and the ones who are involved in the project...because of their commitment. Ah, and because of the financial benefit given by the government (…)” (PU 90-93)

“From the organisation point of view- Total support in this process is important.” (BGU 69)

“Hospital buy - in is very important. If they sabotage the idea then it will not be successful. Peer support is a new way of working that has not been established in civil service, it has no structure in terms of organogram... It doesn’t even have a budget not funded by the government it doesn’t feature anywhere. So it’s a new way of working that very much needs hospital management support.” (BU 12)

“So that is the next problem. You have to prepare the peer support workers for the system, I have to prepare the system for the peer support workers ... And we still have to work well together, because we are actually, I would call it a family, a functioning family. A system that works reasonably well.” (ULM 166)

“Before we talk about peer support, there is need to be a culture of service user involvement; supporting service user involvement in things like co-production, task shifting, task sharing, where service users are recognized as people who are useful resource that can help in bridging the gap between the traditional biomedical model and the recovery model. So service user involvement comes first before you think of peer support, by having service users involved in the treatment and care of their peers in the hospital. So service user involvement is the foundation of peer support; recognizing the useful untapped resources that the service users have got like peer knowledge and experience and harness it (…) this has to be embraced before thinking of peer support per say; without this it will not be on a firm foundation; it will be on a shaky ground and it will not be recognized.” (BU 15-16)

“(..)To what extent does an institution open up not only to an additional employee, but that it really is a different perspective? So how do you let that get on your team?” (UKE 26)

“Well, I think there are few who know a little bit about the district hospital 1 and know a little bit about individual sections, I think they know which wards are more open than others. So ... And it's often up to ward managers and then the staff in the team.” (ULM 14)

“First of all, there has to be a willingness to take in this change of perspective. That means a department of psychiatry, a medical, healing department, has to follow the assumption that there is something else like medicine. And if ... As soon as a physician, whether it's a senior physician or a nurse, has the idea that they are the only ones who know how to do it, and they know what mental illness is, there is no chance of getting anyone in. That's one side of being, I will call it authority. Because, it's a powerful position. And the other side is that the people who are supposed to do this, the so-called experienced people, have to be enabled to... act.” (ULM 26)

“As we PSW should be aware of the values and norms of [Name of organization] this can be done during the training as something extra so that we can comply with the culture at [Name of organization]. So when we are at [Name of organization] we should live as the people of [Name of organization], we should be taught about the norms and values at [Name of organization] so that we can comply with them (…) we should not interfere with their norms and comply with them. For example the mental health workers are the ones who teach about stigma to people with mental illness but they are the ones who practice it, so we can see that but we should not practice it, we should comply with the discrimination policy.” (DS 73)

“(…) What people are here? Can I find people who fit in? Well, we here at the site are not currently recruiting peer support workers. Unless we know we're going to fit. So, there's no job opening where you can apply for and then you have to take someone. We need people that we can get along with. Otherwise it's not going to work. Because then we have so much internal friction that no one outside can benefit.” (ULM 162)

“Of course if the hospital buys the idea, then it will be sustained. But if PSWs do not comply with hospital rules and regulations, then they will always be medicated and discharged and will not be recognized as people of value. During the previous Brain Gain project, there have been incidences where PSWs have sued the hospital to courts of law in regard with the use of seclusion rooms. Such can easily make the hospital loose interest in working with PSWs. It’s better to come at a round table and agree.” (BU 42)

## ***Post-intervention FGs (CFIR Domain 3: Inner setting)***

“I got to speak to X [Name of employee], about things that could come up, and these things did not happen at all in a branch that is ready for it, that speaks the language, where this is understood, I think it is very very important for an easier implementation.” (BGU 21)

“I strongly relate to what P4 [FG participant] said earlier about how the branch is… how much it holds the agenda and carries the importance [of peer support work].” (BGU 41)

“I don’t think it is in that level but really we’ve been sitting here now and look how we talked more than an hour about the program [UPSIDES], I can’t say it in ‘pixels’ like P3 [FG participant] can, but I know how much “buzz” was around it, how present it is, over all (…).” (BGU 147)

“P1:I very much agree with what P2 [FG participant] is saying, I think it gave us the ability to distinct – what is the role of a PSW comparing with a person that comes with a life experience and chooses whether to share it or bring it into their work and as long as… I see what X [PSW] is doing and we have things being done, UPSIDES is one of them and it is really very meaningful, it allowed us, and still does, to make the perception we want in the organization present, and that is really… it might not be the main purpose of the intervention but it definitely supports this agenda as well, this is also an organization of service users in the end, and its growth and its ability to bring other parts into chosen places, for us it was taking advantage of this place for the perception we want to establish in the organization.

P2: I join everything P1 [FG participant] (…) say, that is, we looked at it as a perception, an agenda, something we want to see in our organization in a fundamental way.” (BGU 54-55)

“(….) what I mean is considering peer specialists [PSWs] within the organization… what is the meaning of it, what are the advantages and disadvantages, it helped us a lot with the thinking.” (BGU 112)

“(…) people [PSWs) are not assigned to the ward and that they are separated from the teams instead.” (ULM 27)

“(…) the peers in [hospital 1] are not even in the system, they run separately. Now the UPSIDES peers run separately, they don't interfere with the system. But at the moment when they [PSWs] are, so to speak, outside (…) And I believe that if UPSIDES could have done something, then it would have been, I don't know, to make it more public. But UPSIDES is not the first, so to speak, the first element that is trying to break through in [hospital 1].” (UKE 103)

“In peer work in general: which model do I use? Do I take this isolated solution with a counselling office somewhere or do I really, and this is always my intention, please take it into the multi-professional team? And I think that in order to initiate a change within an institution, within a ward, within a team, or also really this reflection, as P3 [name of FG participant] said. Because I think it's easier to deal with this in a multi-professional team than in an isolated solution. Because in the isolated solution, yes, there is an office somewhere at the end of the corridor and yes, he is allowed to work there. But when I am in the team, when I am in the team meeting, when I am in supervision. When I'm in the intervision. And I can bring my feelings, my perceptions or my points of view into it, I can reach other colleagues somewhere more easily than if I have my office somewhere far away. And as P 3 [Name of FG participant] said so well: he doesn't disturb my system.” (UKE 115)

“The effect on the professionals is a minimal expansion of diversity of perspective, tolerance, liberality or openness, openness to diversity of perspective (…).” (ULM 65)

“I: (…) Has UPSIDES contributed to any changes at District Hospital 1? And if so, which ones? Were there any changes here at the site due to UPSIDES?

P1: No, I don't think so.

P2: I'd say so.

P3: It was too little intense and too little frequent for that.

P2: Not so frequently, I would also say, yes. And I think the difficulty, to the previous question, was really such negative experiences with peer support and that kind of thing is doing the rounds and then colleagues are already sceptical when anything new comes along, yes. I think that made it a bit difficult as well. At the beginning at least. They [MHWs] have already noticed that it [UPSIDES] runs quite differently and so on. But I also didn't notice that anything had changed.” (ULM 78-87)

“(…) I get the feedback from the wards that the offer is basically not used enough. I think there is still a lack of information from the teams on the wards. I can't quite explain it, because we have really made several attempts within the framework of the project and have somehow undertaken several actions, up to the point that in our ward in the conference, the UPSIDES peer support workers were on site, have also repeatedly presented, have repeatedly given material to the hand, which was carried to the other wards, what are the inclusion criteria, what are the exclusion criteria? How do you initiate the encounter between a peer support worker and a patient, how do you initiate that? But my impression is that the offer could still be used more and better.” (ULM 25)

“I was on two wards and asked my colleagues, do you know someone who would be suitable for this project? And I have to explain everything to them again, what it is all about and there is nothing left to do but to go there again and again and ask, I think this general introduction in front of the ward round or in front of the patients is already good, but to ask the staff on the ward, which patient can we address specifically? To whom can I introduce this specifically? I think that could be more effective (…) So it's not quite in people's heads yet.” (ULM 27)

“I: (..) Has UPSIDES contributed to changes in your organization? If yes, which changes?

P1: I can say that officially it has not brought any changes because we are still in the implementation process, I think that when we come to the end then we will come back here at the department and describe to them of the things which happened, explain to them about the results and discuss about how the peer support intervention can be incorporated in the institution and be one of their programs or services which we are providing." (DS 83-84)

“Implementation process was a success (…) in my opinion, if we work on that, UPSIDES peer support in the next two years will be an excellent project because our participants have proved to us that it really worked for them (…).” (BU 60)

“Personally, I have seen that it is successful because we haven’t heard of complaints regarding the project and many things which I am seeing are positive, through the peer support workers the service users are happy. I haven’t seen any challenge, I think it has been successful the way it has been implemented. (DS 109)

“I don’t have any evidence to say that the implementation was not successful, challenges do exist but it doesn’t mean that it has not been successful.” (DS 126)

“And also what went well is the way the communities, families and the hospital staff embraced the project …, It has been well and the credit goes to the project …, the management and the coordination because people really embraced it. And I wouldn’t say there was much sensitization; but am impressed by the way people are understanding the project and…, how they are welcoming the peer support work; is also a surprise and so promising.” (BU 19)

“Engaging health care workers has also been easy because we were also involved in the project.” (DS 64)

# **Service users, mental health workers and peer support workers (CFIR Domain 4: Individuals involved)**

## ***Pre-intervention FGs (CFIR Domain 4: Individuals involved)***

“I was thinking that spiritual beliefs some families might not support PSW as they believe mental illness can be treated spiritually.” (DS 84)

“(…) if we will be going to the community what modality is set for us to be accepted while we are there bearing in mind that mental illness is kept as a secret that people don’t like it to be exposed (DS 25)

“There is a judicial relationship between the service provider and peer support worker which makes it complicated to keep boundaries. A service user can easily make mistakes but because he has history of mental illness; he cannot be reprimanded. I anticipate such challenges to occur.” (BU 23)

“P1: There is likely to be a challenge of work ethics, in a way that there is need for supervision on how PSWs are relating with service users. If it’s violated what happens?

P2: There is likely to be a challenge on how responsible or accountable can service users be. How they could be accountable and yet give them information and tasks to handle” (BU 24)

“P3: When PSWs get a relapse they demoralize others. Service users will think that recovery is not possible because they will say if so and so can get a relapse, then there is no recovery he has been preaching it but now he is also admitted.” (BU 21)

“There is the issue of dealing with breakdowns. One of the mentors has six service users, and is very professional and there was a period of time he [PSWs from other peer support project] was hospitalised for two months it requires organisational preparedness. With a second mentor. We had a plan between us. What she would require if a breakdown would occur. It requires extra preparedness. From what I've seen they are really great at what they do.” (BGU 44)

“Another difficulty will be from the mental health workers, they might not accept PSW and might not show support to PSW in a crisis.” (DS 69)

“I have already noticed that there are also negative attitudes from staff. And I think we need areas where good experiences are made. So that PSW don't get worn out right away.” (ULM 10)

“Well, that's more than I thought. I'm just a little concerned, let's just say. That there might be too many reservations from the start. And that's the way I feel about it. That it could possibly fail because of that. And that would be a great pity. That is why I think it would be ... I also think it would probably be important to really think carefully about which ward it [peer support] could be.” (ULM 18)

“In District Hospital 1, and I'm just a bit worried. Let's just say... That you can also go under the wheels. Because there's no welcoming culture. A welcoming culture that says ‘great peer support workers, that's what we need here.’ I wouldn't put it past them and that's important. It's not generally the case. It seems to me.” (ULM 86)

“With regards to the mental health workers to some extent they might see it as a threat to their employment. They might see that some of their roles will be taken by peer support workers.” (DS 54)

“And what I also notice again and again are the fears of the employees: Is he taking away my work now? Do I have an additional client? Or, or. The fact that if you bring in peers into existing teams in advance, you also discuss this with the team and work on it so that it is known.” (UKE 33)

“We meet a lot of people in the wards who come back all the time. I've been in district hospital 1 for five years now and there are a lot of patients who are always coming back. And I believe that ... that changes our view of mental illness. And I believe that you have to... you have to be aware of that. That's why employees who encounter a peer support worker probably live with this fear from the start, because nobody can really get back into life. And this is a wrong point of view, but I think that's what happens when you are in this system (ULM 55)

“(..) the danger (…) [for MHW is], do I have a new client? Or the danger: He [the PSW] can do anything now.” (UKE 39)

## ***Post-intervention FGs (CFIR Domain 4: Individuals involved)***

“I can say that I saw the service users when they left the group and one by one the approached me and said, ‘wow it was really good, you should bring more of those’, ‘so great we had this’, ‘they treated me with respect and it was so important, and I received so much for my life’, it was very significant for the facilitators.”(BGU 146)

“Majority of the service users received the service well, they need people to visit them and talk to, they find it as comfort to have people who visit them and this person is also a person with lived experience of mental illness and not a profession who has never suffered from a mental illness but talks about it.” (DS 33)

“Let’s talk about the relatives of the users, I think they were ready and they saw that it is something which helps them, they saw that it was sufficient help for them by having UPSIDES peer support because it brought them to the health care providers and peer support workers, they could easily communicate if there was any challenge.” (DS 100)

“Additionally, it has been a demand, relatives are calling us and asking when the PSW will go to visit them because they have helped them a lot, nowadays they don’t force the services users to go to the clinic they go on their own. So we are seeing that the community wish this service to continue to be provided because it has seen benefits from their patients. Apart from the patients even relatives say that UPSIDES peer support has helped them a lot.” (DS 121)

“And then some families were actually not welcoming because they had their own perception on what mental health is…, but at least we got a way of handling that much but it didn’t come an easy way… but I think it was…, we interested them.” (BU 39)

“It is just the cooperation and there are some who gave transport fares to the service users. Also the issue of acceptance because some of them are wives, others are husbands, so accepting the patient to be in the program and come here to attend the sessions because there are others who had refused their relatives to come for the intervention sessions and had refused to visit them at home. So I think that most of them were given cooperation.” (DS 102)

“The biggest challenge from the service users is that they were not cooperating with the peer support workers, sometimes when the PSW call them they say that they are not around, so in the beginning they lacked cooperation.” (DS 57)

“P3: (…) what I (…) also experience now that you are company people that people also cancel (…) and it's easier to tell a peer than a professional, the question is how seriously do you take it? A peer is a bit... So, do you postpone faster? Or don't get in touch at all?” (UKE 184-188)

“There are some clients whom we were linking to the study but they didn’t have time to come, sometimes they say that there is a challenge with transport fare. You find that there are others who are interested but the issue of time is a challenge.” (DS 23)

“(…) as per my observation, the public that we have on the OPD basis is a very general public. They are very unknown about this project. So I feel like they are more in need. As far as I fill the forms,. that is how I understood.” (PU 81)

“Apart from receiving health education some of the service users saw it as a room to establish relationships, it is something which I saw as a challenge. Majority of the service users received the service well, they need people to visit them and talk to, they find it as comfort to have people who visit them and this person is also a person with lived experience of mental illness and not a profession who has never suffered from a mental illness but talks about it.” (DS 33)

“The biggest challenge on the part of the service users is going beyond their boundaries, they begin to annoy the peer support workers there are some service users who want to establish romantic relationships so the PSW become afraid of visiting them in their homes as it might be risky to them especially women. The PSW sees that it is a risk to go and visit the service user so it is a challenge. The PSW is afraid and the service user goes beyond his boundaries.” (DS 55)

“So maybe after this, you know, they can have a vocational rehabilitation because you know, they have already started working here as part of the staff although they are volunteers and we are not giving them huge incentives.” (PU 27)

“The surprise I got mostly was from PSWs…, because I realized that they were very committed and they had patience in whatever they were doing (…) I really got surprised that people never gave up. Then also another one is about the PSWs that were trained …, still connected to patience and liking the work that they are doing (…)” (BU 24)

**“**What surprised me is during this covid19 pandemic.., when we had a lockdown. The way these PSWs were so concerned about their medication refills, how to keep well, how to maintain their wellness, how to prevent the relapses.., was so surprising; meaning that they owned ….; and not only were they concerned about their wellness but also some of them went an extra mile to getting concerned of how their peers could survive in the lockdown. The way they could call…, giving directions about their peers medication lists …, how far they’ve gone, how many pills are remaining with, how they would miss out in case they don’t get the supplies..., was really a surprise. So I was surprised of their commitment…, and the need to help…, and the way they loved working as PSWs. (BU 25)

“First of all, I might be surprised by the peer support workers, the way they took up that job you can see that they are in the mood to do the work.” (DS 34)

“Also between themselves the PSW established their network and they used to make follow up on each other, for example one of them had relapsed they made phone calls and went to his home. So within the PSWs themselves they saw that a certain person has changed so they should support each other, so their network enabled them to be close to each other.” (DS 37)

“This [the implementation] has allowed a real togetherness of the peers with each other and with the clients (…) from my point of view the UPSIDES team grew, stayed together and that was not the case with the peer support experiences in the psychiatry clinic before.” (ULM 21)

“My overall opinion on this, I see so far the intervention as a success and with this I believe the team is committed. I also view the interest and experience of the PSWs …, that they are interested and where they are finding a challenge, they do consult. This has helped in limiting the failure hence intervention being a success for now.” (BU 11)

“I also think that the people who do this peer work now put themselves under relatively strong pressure.” (UKE 22)

“You burn yourself out. Because in most places you are alone at the location. Alone against twenty, thirty colleagues. I am in the team (…) I have professional colleagues or experts from teaching all around me and I am the only one as an expert from experience and that always feels like a wall for me when I address such topics.” (UKE 82)

“I would also want to add a voice that UPSIDES has brought about awareness to mental health teams and also to the managers about the importance of engaging PSWs in mental health care…” (BU 32)

“P1: The changes which have been contributed by UPSIDES peer support, sometimes health care providers have negative attitudes, so UPSIDS has enabled to change attitudes among health care providers, the attitudes which we had on our clients.

Facilitator: How? Can you give briefs details?

P1: In the past we might have had attitudes that it is not possible for the client to be recover or be stable, but after being enrolled to the project you see this person is stable, so you change your attitudes on the way you are caring for the patient. I think there are changes in attitude on patients, the way they were before the project started and when they were enrolled in the project they changed, so out attitudes have changed.” (DS 87-89)

“(..)PSVs [peer support volunteers] are currently involved in 5-6 activities, highly involved. (…) In the ward, communication related to recovery-oriented planning is their responsibility. Plus, scheduled activities with the patients in the ward like yoga, prayer, meditation in the morning is being conducted by ward based PSVs.”(PU 4)

“(..) there have been what we call peer supervisions where PSWs come together to share challenges and to support each other…, this has been very key in Butabika site..., it has helped other members to work better…, and then they have a forum where they can be supported and also support each other in case of any challenges. But also dining together with them, having those meals together (…) I think these meals mean a lot ..., apart from getting satisfied…, but I think dining with other staff, and peers…, I think it’s something that helped them move on. (BU 45)

“Some of the changes that have taken place is that at least now PSWs are taken as part of the mental health team because the incharges [persons in charge] for example when they see these PSWs come on ward to check on their peers, they do recognize them as people that are going to offer support to these patients.” (BU 50)

“(…) the peers in [hospital 1] are not even in the system, they run separately. Now the UPSIDES peers run separately, they don't interfere with the system. But at the moment when they are, so to speak, outside (…).” (UKE 103)

“(…) Positive from my point of view, because it [UPSIDES] was detached from the clinic. Negative because this per se is an inherent weakness. So positive because it was avoided to immediately tie the UPSIDES implementation closely to teams or teamwork in any way and to connect it to the teams. This has allowed a real togetherness of the peers with each other and with the clients. But this brings with it the weakness that the impact, the effect on the clinic, on the way psychiatry is practiced and on the way of thinking remains limited because what is offered is flown in, is on the outside.” (ULM 21)

“What was of course also a pity, or what I also find a pity, was that there were relatively few inpatient clients, i.e. what I have now noticed, because as a result there was also little contact from the wards to the peers.” (ULM 39)

“And also what went well is the way the communities, families and the hospital staff embraced the project …, It has been well and the credit goes to the project …, the management and the coordination because people really embraced it. And I wouldn’t say there was much sensitization; but am impressed by the way people are understanding the project and…, how they are welcoming the peer support work; is also a surprise and so promising.” (BU 19)

“One of the factors that led to successful implementation of UPSIDES is hospital buy-in which was important because when the project was proposed to the hospital and …, all staff embraced it starting from managers up to the lower levels at ward levels (…) Nurses on wards, Social Workers, Occupational Therapists, Psychologists, Clinicians …, they accepted the idea because they knew it was of importance to mental health care. So all that led to successful implementation of the project.” (BU 41)

“I strongly relate to what P4 [FG participant] said earlier about how the branch is… how much it holds the agenda and carries the importance [of peer support work]. How it was, at our place it was very clear to me that the success of it is in favour of those two specific directors – P3 [FG participant] and [name of another director]. It is clear to me it has much great value.” (BGU 41)

# **Training (CFIR Domain 5: Implementation process)**

## ***Pre-intervention FGs (CFIR Domain 5: Implementation process)***

“(…) so helping people [PSWs] to do that. Before that, you have to plan out how they will do that, we have to train them.” (PU 122)

“Once we have a worker with knowledge from experience we are required to give them guidance which is appropriate so he can see the needs of the service user.” (BGU 32)

“Initially more guidance, maybe give them a mentor, and add caution when assigning them to whom is receiving and whom is giving the service. Sometimes it doesn't work.. because of past traumas.. That also has to be taken into account and also initially space as far as the position is concerned.. Sometimes ‘let’s get started’ doesn't work. It requires further consideration.” (BGU 36)

“They [people with lived experience] have a treasure, namely experience. But just because I have a treasure, I am far from being able to deal with it in a business and economic sense. Just because I have experience, I am far from being able to accomplish anything. And there you should be able to reflect so much about yourself, what can I do, what should I do, what is my mission. Otherwise people run into a very, very sharp knife. That is our experience from where I am. That peer support is actually a great thing, but since it is an institution, peer support workers have a change of perspective from client to employee. And that's a difficult thing. You have to teach it to people. What are you doing now? You're doing business.” (ULM 26)

“(…) we already said is that we are a little pessimistic about going to the community and doing the thing. So the one idea in my mind is that let’s start the same work in the hospital (...) Before we start in the community.” (PU 34-35)

“(…) sustaining is actually not a cup of tea for the supervisor who is actually mentoring the PSVs [peer support volunteers], right? Yes but first is their training - the frequent training that we give them, refresher training (…)” (PU 89)

“I think it is very important from our little experience that even if you are already in a field of work no matter if it is an internship or paid the modules that you have taken up will continue. That you can also look again what is missing (…) So that there is also such practical accompanying training (UKE 14)

“And I think maybe also in the preparation of the peers, so in an extra module or whatever, that you have to give them this feeling that they are also strengthened by you. Because if they are so insecure and then go into a conversation with a client or whatever you want to call them, it is not necessarily profitable for the (laughing) person seeking help or support, ne. That you have to empower them so that they can openly approach the others. And when they themselves have so many fears and so many uncertainties, (UKE 51)

“I've never noticed so much as here how much I have to pay attention to my mental hygiene. And so ... And I think that a peer support employee who simply brings a burden with him in this field must be even more aware of this. (…) What is my task? What can I bring to the table, and what is totally helpful and valuable that I bring it to the table? And where do I have my limits. And then I have to give it to somebody else.”(ULM 299)

“You [research worker in UPSIDES] should prepare some notes about advance directive - what it is, the process, the benefits and how you will make people aware. That kind of notes. Then you will have to train particular PSVs [peer support volunteers] so they can impart this knowledge. If they have this knowledge then they can impart it right.” (PU 127)

*“*Training of peer support workers by giving them the basics on how to handle fellow patients. This will give them the skills to be imparted on to the peers by giving guidelines on what they are supposed to do.” (BU 11)

“I am thinking about the training, PSW should have an equal knowledge on mental illness and how they are to support service users to avoid mixed messages to the society.” (DS 7)

“As we PSW should be aware of the values and norms of [Name of organization] this can be done during the training as something extra so that we can comply with the culture at [Name of organization]. So when we are at [Name of organization] we should live as the people of [Name of organization], we should be taught about the norms and values at [Name of organization] so that we can comply with them.” (DS 73)

“Every mental health professional who takes specific roles and responsibilities, should be trained on one particular topic instead of giving them the whole module. A brief of module, the perspective of module, the main objectives and the role of particular mental health professional for example, psychiatrist. So what is the role of psychiatrists with the PSVs [peer support volunteers]? Right? So we can explain them and tell them the page number. We can also give them the handouts of the pages they are supposed to fill (…) Another thing is...about nursing professionals, right? They must be a large quantity of nursing professionals and they will also be the first contact person. So what is their role? Brief about the PSVs and PSVs’ modules’ and their recovery oriented care plan and their role and responsibilities. Similarly, there are other mental health professionals as well. So, categorise them into groups and train them. This might be beneficial to the PSV, hospital and the mental health professional too.” (PU 84-86)

“I think it needs to happen.., the manager has to have a very strong belief in it, and that will rub off onto the rest of the team. If initially there isn't the will that will affect the rest of the team. I'm accompanied by Consumers as providers program for managers. One of the things I learned and struggled with implementing is to allow my staff to discuss their challenges with this, although I’m learning. I had 3 coordinators and one of them has trouble with this and he understood that there was no choice. He was in a situation that he had no choice over and accepted it in a positive way.” (BGU 30)

“And so I just think we have to talk, talk, talk. We have to get into the teams and the peer support workers have to get into the teams and have to make themselves known. And so that the others develop a sense of how valuable this can be and will be.” (ULM 56)

“If you are already integrated into existing teams it is important to clearly train the team, the professional team, beforehand and to let them develop an attitude. Just really going into a recover really goes in a direction that neither the team nor the peer is then burned in the end. So quite clearly: because attitude cannot be prescribed, it has to be developed. There is an incredible amount of preparatory work within a team before you put a peer on that team. Even if he's in a staff meeting or something (…) I think it would be good and perhaps also important, to establish contact between the institution and the peer before the actual work takes place.” (UKE 33)

*“*It’s important to allow other stakeholders to learn about what peer support is by spreading it all over like a catch fire for everyone to know about it. In such a case, everyone in the hospital should be able to be sensitized about UPSIDES peer support. Otherwise if they are not aware, then they will not cooperate.” (BU 14)

“And so I just think we have to talk, talk, talk. We have to get into the teams and the peer support workers have to get into the teams and have to make themselves known. And so that the others develop a sense of how valuable this can be and will be.” (ULM 56)

“It needs to come from us [mangers]. Encouragement.. explaining.. and explaining how much it can benefit. A WhatsApp message just isn't enough. If we explain the significance and importance.. it will work better. To explain the opportunity. The opportunity to get a response.. A different response“ (BGU 50)

It's clear to me that some of the management coordinators will spread the message and some won't. What's important to me I will take care of. Every day I want to know who you spoke about it to.. Then the team understands.. that's important to me.. The personal involvement.. I as a manager want to know why no one is interested etc. We need to be on top of this.. Continuously go back to being the manager to move it forward. (BGU 52)

“I think it is the management, it needs courage for the management to accept a new thing and not only the management but any change which takes place somewhere it is difficulty to fully accept it or allow it to operate without knowing or having assurance of the impact or outcome. (…) So the management will have difficulties in deciding, when they decide the service providers will just accept, they will be given the terms and tools they have to implement.” (DS 68)

## ***Post-intervention FGs (CFIR Domain 5: Implementation process)***

“One of the factors that made it most successful was the trainings of both the PSWs and the staff because it helped them to have enough knowledge on how to implement what they are supposed to do..., and also our RAs were able to reach the community and were able to identify any mental health problems and this helped patients to be helped earlier before relapses came in.” (BU 36)

“I think that the implementation was not bad, it was conducted well considering the fact that the peer support workers received training about which things they are supposed to do, which ones they are not supposed to do, how they can meet with their service users and if they face a challenges they should involve staff or responsible people in the project. So, the way the project was implemented I think that it was implemented well (…)” (DS 11)

“As my colleague said, the implementation process went well by training peer support workers who were delivering the intervention to the service users. What is more important is that the peer support workers were able to interact with the families of those service users to the extent that they were well accepted, they were accepted by the relatives because of good preparations.” (DS 12)

“The other thing is that I think the training which peer support workers received enabled them to perform their roles as they used the knowledge which they received in educating the services users.” (DS 16)

“I think the first thing is the training which was given to the peer support workers, this was very important because I don’t think that they would have been able to do what they were doing without having that package of all the things which they were taught, that is very essential.” (DS 62)

“I think the workshop and training to the peer support workers enabled to strengthen them. I think the training helps because this person was facing stigma of his illness, maybe the community rejected him before so if he doesn’t receive training it might be a challenge. So in order to implement this project properly a workshop or training should be conducted so that the PSWs can know about their roles. Engaging health care workers has also been easy because we were also involved in the project.” (DS 64)

Then we did a second course, which I found too tight - too tight in terms of time. Not at all with the modules. For me, the implementation problem is not that there are not enough modules. It's that I think the time intervals between the modules should be much longer (…)I also think that the people who do this peer work now put themselves under relatively strong pressure. (UKE 22)

“I could have done this somewhat more practical, or perhaps - I don't even know what to call it - it wasn't more unintellectual either. But something is more direct, more immediate in the UPSIDES thing, I think. Even just the fact that there is this booklet. This booklet has something very, very practical and something that people can use when they want to follow something, where there is a structure, not in the sense of: you have to do it this way that was never said that way, but something that helps me, that can somehow also centre me a bit. And I actually found that good.” (UKE 49)

“And I think that UPSIDES should take half a year, three quarters of a year, as [speaker 3] has already suggested, as a training or further training period, and when Ex-in is added, that would be what you would have to saddle on top of it, plus additional modules or some kind of recognition year.” (UKE 67)

“According to me, we can think about how to involve PSVs [peer support volunteers] more. I think, we need to empower them more...If they are empowered more, like P2 [participant 2] said, their confidence has built up already (…) we should train them more on coping mechanisms which we already do but it needs to be done in a more systematic way. So, I think, if they are more empowered the quality can improve further. And what is but obvious is capacity building is must. Every 3-4 months, if we keep a review meeting or refresher training, it will be much more beneficial to the hospital and the PSVs both.” (PU 61)

“I would also want to add a voice that UPSIDES has brought about awareness to mental health teams and also to the managers about the importance of engaging PSWs in mental health care…, because before UPSIDES actually started off; there are so many activities that took place …, we had workshops on organizational preparedness plan where we made sure we held workshops and sensitize people about the upcoming project which engages using peer support and as a way of developing empowering mental health services. So most of the managers were able to come to understand about the importance of peer support.” (BU 32)

“Implementation process was a success although it would have made more…, a bigger percentage if there was continuous conferences. There are transfers from other hospitals or recruitment of new staff..., so continuous sensitization is important so that all ward managers are aware. Also the people engaged in UPSIDES need a clear identification…, because the ward brings in very many people, you cannot identify who is who and what talks about them. Otherwise in my opinion, if we work on that, UPSIDES peer support in the next two years will be an excellent project because our participants have proved to us that it really worked for them…, so if we extend it to even the other people and peers, we will be good to go.” (BU 60)

“(…) so… we held a few designated staff meetings for it, we had one with L [employee] about experience-based knowledge as an introduction, we had a joint meeting with the Secondtown [UPSIDE implementation site 2 in Israel] team and the Firsttown [UPSIDES implementation site 2 in Israel] team, a long meeting with the groups that were mixed and divided and talked more specifically about UPSIDES and more specifically about what it means people with experience-based knowledge, how you share it and how you work with it etc. (…) it [is] worthwhile – more time with the teams, in meetings, updates, in any possible way. It is not a team that works inside the branch, so it makes a difference… holding it all the time.” (BGU 48)

“(…) we have really made several attempts within the framework of the project and have somehow undertaken several actions, up to the point that in our ward in the conference, the UPSIDES peer support workers were on site, have also repeatedly presented, have repeatedly given material to the hand, which was carried to the other wards, what are the inclusion criteria, what are the exclusion criteria? How do you initiate the encounter between a peer support worker and a patient, how do you initiate that?” (ULM 25)
