# Supplementary material for: Perspectives of key informants before and after implementing UPSIDES peer support in mental health services: qualitative findings from an international multi-site study
Source: BMC Health Serv Res. 2024 Feb 1;24:159. doi: 10.1186/s12913-024-10543-w (PMC10835950; doi:10.1186/s12913-024-10543-w)
Supplement: Supplementary file 4 — Additional file 4: Consolidated criteria for reporting qualitative studies (COREQ) 32-item checklist [file 12913_2024_10543_MOESM4_ESM.docx]

**Additional file 4: Consolidated criteria for reporting qualitative studies (COREQ) 32-item checklist**

**Domain 1: Research team and reflexivity**

*Personal Characteristics*

1. Interviewer/facilitator: Which author/s conducted the interview or focus group? Two research workers form every site facilitated the FGDs (in total 12 persons).

2. Credentials: What were the researcher’s credentials? E.g. PhD, MD Research workers had mixed backgrounds, including study degrees (bachelor, master).

3. Occupation: What was their occupation at the time of the study? FGD facilitators were research workers in the UPSIDES project.

4. Gender: Was the researcher male or female? Most of the researchers were female, some male.

5. Experience and training: What experience or training did the researcher have? Online training sessions for interviewers to introduce the topic guides were conducted, potential challenges during the FGDs were discussed. Researchers who analysed the data had several years of experience with qualitative research.

*Relationship with participants*

6. Relationship established: Was a relationship established prior to study commencement? Yes, in in most study sites interviewer were known as part of the UPSIDES project before the start of the FGDs.

7. Participant knowledge of the interviewer: What did the participants know about the researcher? e.g. personal goals, reasons for doing the research. Participants had mixed knowledge on the interviewers, depending on whether or not they knew the interviewers and the project beforehand. In all study sites the interviewers introduced themselves, the study and the study aim before the start of the FGD.

8. Interviewer characteristics: What characteristics were reported about the interviewer/facilitator? e.g. Bias, assumptions, reasons and interests in the research topic. Personal characteristics about the interviewers were not reported.

**Domain 2: Study design**

*Theoretical framework*

9. Methodological orientation and Theory: What methodological orientation was stated to underpin the study? e.g. grounded theory, discourse analysis, ethnography, phenomenology, content analysis Transcripts were analysed using qualitative content analysis.

*Participant selection*

10. Sampling: How were participants selected? e.g. purposive, convenience, consecutive, snowball A purposive sample strategy was used.

11. Method of approach: How were participants approached? e.g. face-to-face, telephone, mail, email Participants were contacted face-to-face, telephone and email.

12. Sample size: How many participants were in the study? 54.

13. Non-participation: How many people refused to participate or dropped out? Reasons? Several participants dropped out due time constraints.

*Setting*

14. Setting of data collection: Where was the data collected? e.g. home, clinic, workplace FGDs were mainly conducted in the organisations where UPSIDES was implemented (e.g. hospital).

15. Presence of non-participants: Was anyone else present besides the participants and researchers? No.

16. Description of sample: What are the important characteristics of the sample? e.g. demographic data, date. Please see table 2 and 3 in the appendix.

*Data collection*

17. Interview guide: Were questions, prompts, guides provided by the authors? Was it pilot tested? Semi-structured topic guide were used. One test interview was conducted.

18. Repeat interviews: Were repeat interviews carried out? If yes, how many? No.

19. Audio/visual recording: Did the research use audio or visual recording to collect the data? Audio recording was used.

20. Field notes: Were field notes made during and/or after the interview or focus group? Yes.

21. Duration: What was the duration of the interviews or focus group? 30 to 105 minutes.

22. Data saturation: Was data saturation discussed? Yes, we assumed that sufficient saturation is achieved by conducting 2 FGDs per study site, resulting in a total of 12 FGDs.

23. Transcripts returned: Were transcripts returned to participants for comment and/or correction? No.

**Domain 3: Analysis and findings**

*Data analysis*

24. Number of data coders: How many data coders coded the data? Four

25. Description of the coding tree: Did authors provide a description of the coding tree? CFIR Domains were described.

26. Derivation of themes: Were themes identified in advance or derived from the data? Both ways.

27. Software: What software, if applicable, was used to manage the data? MAXQDA 2020

28. Participant checking: Did participants provide feedback on the findings? No

*Reporting*

29. Quotations presented: Were participant quotations presented to illustrate the themes / findings? All themes are supported by quotes, which can be found in the appendix. Some quotations can be found in the results. Was each quotation identified? e.g. participant number Yes

30. Data and findings consistent: Was there consistency between the data presented and the findings? Yes

31. Clarity of major themes: Were major themes clearly presented in the findings? Yes

32. Clarity of minor themes: Is there a description of diverse cases or discussion of minor themes? Minor themes (e.g. few cases in which PSWs received little acceptance from SUs and their relatives) are described and diverse perspective of key informants (e.g. about the organisational benefits of peer support) are discussed
